# Phenotypic, molecular and pathogenic characterization of *Colletotrichum scovillei* infecting *Capsicum* species in Rio de Janeiro, Brazil

**DOI:** 10.7717/peerj.10782

**Published:** 2021-04-27

**Authors:** Renata Mussoi Giacomin, Claudete de Fátima Ruas, Viviane Yumi Baba, Sara Mataroli De Godoy, Claudia Pombo Sudré, Cintia dos Santos Bento, Maura Da Cunha, Ingrid Gaspar Da Costa Geronimo, Rosana Rodrigues, Leandro SA Gonçalves

**Affiliations:** 1 Biology, Universidade Estadual de Londrina, Londrina, Paraná, Brazil; 2 Agronomy, Universidade Estadual de Londrina, Londrina, Paraná, Brazil; 3 Plant Breeding, Universidade Estadual do Norte Fluminense, Campos dos Goytacazes, Rio de Janeiro, Brazil; 4 Department of Agronomy, Universidade Estadual de Londrina, Londrina, Paraná, Brazil

**Keywords:** Multilocus sequencing, Molecular species identification, Genetic diversity, Pepper anthracnose, Capsicum–Colletotrichum interaction

## Abstract

Anthracnose is a disease caused by *Colletotrichum* spp., one of the world’s most damaging sweet and chili pepper pathogens, especially in tropical and subtropical regions. In the state of Rio de Janeiro, anthracnose is one of the main obstacles for pepper crops. However, to date no research has focused on the identification and characterization of the pathogen, which is fundamental to understand the scope of the disease in the state. Thus, the correct identification of the fungal species and pathogenicity studies can provide important support for disease management and control, apart from identifying possible resistance sources for exploitation in peppers breeding programs. In this study, 11 *Colletotrichum* isolates were collected from peppers with typical symptoms in the Rio de Janeiro state. These isolates were characterized based on morpho-cultural characteristics and sequencing data from five regions (ITS, ACT, CAL, β-TUB and GAPDH), and the genetic variability was estimated by AFLP markers. Simultaneously, microscopy images of the colonization by the fungal species on unripe *Capsicum annuum* fruits were taken. Pathogenicity was tested and resistance sources were sought by means of infection of ripe and unripe fruits of 50 *Capsicum baccatum* accessions. The resulting data showed that all isolates belong to *Colletotrichum scovillei* specie. About the pathogenicity of *Capsicum baccatum*, differentiated, stage-specific responses, with higher resistance of ripe fruits were recorded. In addition, four possible sources of *Colletotrichum scovillei* resistance were detected among the tested accessions. The combination of these data can contribute to future studies on the interaction of *Colletotrichum scovillei*-*Capsicum* spp., a research line that is still unexploited in the main areas of this anthracnose fungus.

## Introduction

The genus *Colletotrichum* is considered one of the most harmful phytopathogenic fungi in the world, due mainly to its wide range of hosts and level of aggressiveness. Species of this genus can cause diseases on most crops in the world (Dean et al., 2012; Mongkolporn & Taylor, 2018), and are divided phylogenetically into nine major complexes: *Colletotrichum acutatum, C. gloeosporioides, C. boninense, C. graminicola, C. spaethianum, C. destructivum, C. dematium, C. truncatum* and *C. orbiculare* (Cannon et al., 2012).

The identification of *Colletotrichum* species was traditionally based on morphological and cultural characteristics (Cai et al., 2009). However, nowadays it is well-known that is unreliable, since this kind of characteristics is highly influenced by environmental conditions. Thus, it is strongly recommended the application of molecular analysis (Hyde et al., 2009).

Several studies have used an association of phenotypic traits with the application of a multilocus phylogenetic analysis (Sharma et al., 2005; Cai et al., 2009; Hyde et al., 2009; Cannon et al., 2012; Noireung et al., 2012; Weir, Johnston & Damm, 2012; Marin-Felix et al., 2017; Mongkolporn & Taylor, 2018). The first molecular applications to distinguish *Colletotrichum* species were based on the ITS (rDNA) gene sequences in 1992 (Mills, Hodson & Brown, 1992; Sreenivasaprasad, Brown & Mills, 1992). Since then, it is suggested to develop multilocus phylogenetic analyses for the identification of taxa, based on conserved sequences. The currently used gene sequences for the identification of *Colletotrichum* species include: rDNA spacer sequences (ITS), glyceraldehyde-3-phosphatodehydrogenase (GAPDH), actin-like protein (ACT), β-tubulin (β-TUB), glutamine synthetase (GS), calmodulin (CAL), among others (Marin-Felix et al., 2017).

Sweet and chili peppers (*Capsicum* spp.) are vegetable crops that are strongly affected by anthracnose (*Colletotrichum* spp.), mainly in tropical and subtropical regions, where the development conditions for the pathogen are favorable (Roberts, Pernezny & Kucharek, 2001). These vegetables are among the most important constituents of cooking in these countries, mainly because of the versatility of consumption forms and high nutritional value. Brazil produces around 5 million tons year^−1^, but most of this production never reaches the final market. However, in spite of all technological advances in production, anthracnose is still reported as an extremely harmful disease for production in these regions, by causing lesions on the most economically profitable element of the crop, the fruit (Park et al., 2012). Typically, fruit symptoms are characterized by small roundish or angular water-soaked sunken lesions with light brown margins. In the early infection stages, the lesions form concentric rings of moist, usually salmon-colored acervuli (Than et al., 2008).

The etiology of anthracnose-causing *Colletotrichum* species in peppers is highly complex. Until 2009, only three main species (*C. acutatum*, *C. capsici* and *C. gloeosporioides*) were recognized as causative agents of the disease. Later, the taxonomy of *Colletotrichum* was revised, based on multilocus analysis. So far, 24 *Colletotrichum* species were described that can attack peppers, seven of which of the *acutatum* and nine of the *gloeosporioides* complex (Saxena et al., 2016; Mongkolporn & Taylor, 2018). To date, anthracnose symptoms caused by six species have been reported in Brazil: *C. acutatum*, *C. gloeosporioides*, *C. coccodes*, *C. boninense*, *C. capsici* and *C. scovillei*. Until 2009, *C. gloeosporioides* was reported as the predominant species infecting *Capsicum* (Tozze et al., 2009), but, since 2014 that Caires et al. (2014) first reported *C. scovillei* infecting peppers in Brazil, this species has become evident in different regions, like Northeast region (Silva et al., 2017). However, most of these studies were developed based on only morphological and ribosomal DNA internal transcribed spacer region (ITS) analyses (Júnior, Mello & Júnior, 2006; Tozze Júnior et al., 2007; Tozze et al., 2009; Caires et al., 2014).

The correct identification of anthracnose-causing species is the first step towards understanding the pathogen/host relationship and to develop effective control strategies, such as the identification of resistant cultivars and an optimized disease control management. To date, knowledge about the taxonomy and diversity of anthracnose-causing species in Brazil is still limited. Thus, the objectives of this study were: (i) to use multilocus markers and phenotypic traits to identify and characterize *Colletotrichum* species associated with *Capsicum* spp. in chili and sweet pepper crops in the Rio de Janeiro state—Brazil; (ii) To determine the colonization of *Colletotrichum* spp. in *Capsicum annuum* L. by electron microscopy; (iii) investigate the reaction of *Capsicum baccatum* L. to anthracnose (*Colletotrichum* spp.); and (iv) identify possible resistance sources to *Colletotrichum* spp. that can be used in future breeding programs.

## Materials and Methods

### Isolate collection, monosporic cultures and maintenance

The 11 *Colletotrichum* spp. isolates used in this study were collected by staff of the Plant Breeding Laboratory of the Universidade Estadual do Norte Fluminense Darcy Ribeiro (LMGV/UENF) at five different locations in Rio de Janeiro state, from February 2013 to November 2014. The samples were collected from *Capsicum* spp. fruits with typical anthracnose symptoms, characterized by sunken circular spots with concentric rings and masses of orange conidia (Table S1). The fruits were collected separately to avoid cross contamination. The location of each sample are described on Table S1.

Spores were isolated aseptically, with a histological needle, by direct transference from the lesions to Petri dishes containing potato dextrose agar (PDA) medium + streptomycin. To ensure the genetic uniformity of the isolates, monosporic cultures of all isolates were used. For this stage, one ml of mycelium + water suspension was transferred to petri dishes containing 10% Water-Agar medium. The plates were maintained for 24 h at 22 ± 2 °C. Then, germinated conidia were transferred individually to the center of Petri dishes containing BDA culture medium with antibiotic.

The isolates were placed in a growth chamber at 25 °C until mycelial growth (7–10 days) and then stored at 4 °C until use.

### Morphological and cultural characterization

The following morphological characteristics were evaluated: conidia morphology, and characteristics such as culture appearance, color and growth rate. To determine the colony growth rate (CGR) and colony color and appearance, the monosporic isolates of *Colletotrichum* spp. were subcultured by placing mycelial discs (Ø 8 mm) in Petri dishes (Ø 90 mm) containing PDA medium + streptomycin medium. Five replicates per isolate were evaluated for 10 days or until the first colony covered the total plate area. The plates were maintained in a growth chamber at 25 °C under a 12:12 h light:dark photoperiod. Orthogonal measurements were performed every 24 h with a caliper and the mycelial growth rate was expressed in mm/day.

Conidia length and width were measured on images captured by a digital camera coupled with a Zeiss optical microscope, Olympus BX 60, using software ZEN (Zeiss^®^, Jena, Germany). The characteristics evaluated were compared with the descriptions of *Colletotrichum* species proposed by Sutton (Sutton, 1992) and updated by Damm et al. (2012).

### DNA extraction

The 11 *Colletotrichum* spp. isolates selected for the study were incubated on PDA medium for seven days in a growth chamber. The DNA was extracted by scraping the mycelium from the plate with a sterile Drigalski spatula *or* loop. The methodology proposed by Zolan & Pukkila (1986) was used, with modifications. The scraped mycelium was macerated with liquid nitrogen and the extraction buffer containing CTAB (cetyltrimethylammonium bromide, Sigma–Aldrich, St. Louis, MO, USA) was immediately added. The quality of the total DNA was verified by 1% agarose gel electrophoresis. The DNA concentration was estimated with a NanoDrop 2000/2000c spectrophotometer (Thermo Scientific, Waltham, MA, USA).

### Sequencing

The 11 *Colletotrichum* spp. isolates were identified based on multilocus phylogenetic analysis of the genes glyceraldehyde 3-phosphate dehydrogenase (GAPDH), actin (ACT), calmodulin (CAL), β-tubulin (β-TUB), and the rDNA spacer region (ITS-DNAr) (Table S2). The amplification reaction was performed with 25 ng DNA; 7.5 μL Gotaq^®^ Green Master Mix (Promega, Winchester, VA, USA), 10 μM of each primer; 10% DMSO (dimethylsufoxide) and ultrapure water to complete the reaction volume to 15 μL. The cycling parameters to amplify the fragments used in this study were the same as those described by Weir, Johnston & Damm (2012) and Damm et al. (2012). The amplification products were checked on 2% agarose gel. Prior to sequencing, the samples were purified with Illustra ExoProStarTM 1-Step, as recommended by the manufacturer (GE Healthcare, Chicago, IL, USA).

The sequencing reactions were performed in a volume of 10 μL with: 2.3 μL buffer (5×); 10 μM forward primer; 1.0 μL BigDye Terminator Ready Reaction kit (Perkin-Elmer; Applied Biosystems, Foster City, CA, USA); 1.0 μL purified amplification product and completed to 10 μL with ultrapure water. This reaction was repeated and performed separately for the reverse primer. Sequencing was carried out using a BigDye Terminator Cycle Sequencing Ready Reaction kit (Perkin-Elmer; Applied Biosystems, Foster City, CA, USA) and an ABI 3,500 xL Genetic Analyzer (Applied Biosystems, Foster City, CA, USA).

### Data analysis—phylogenetic relationships

The base-calling and assembly of contig sequences were performed with Phred v. 071220.c (Ewing & Green, 1998; Ewing et al., 1998) and Phrap v. 071220.c (Ewing & Green, 1998; Ewing et al., 1998), both with the package Chromaseq v.1.12 (Maddison & Maddison, 2014), and the alignment with software MUSCLE v.3.8.31 (Edgar, 2004), implemented in Align v.1.11 (Maddison, Wheeler & Maddison, 2007). Both Chromaseq and Align are part of the phylogenetic computing system Mesquite v.3.01 (Maddison & Maddison, 2014). Gaps were coded by SeqState v1.4.1 (Müller, 2005), using the simple indel coding (SIC) method (Simmons & Ochoterena, 2000).

Among the 11 isolates only two unique haplotypes were found for the five sequenced regions (see results), only the UEL01 and UEL27 isolates were analyzed together with the sequences from GenBank (Table S3). Bayesian phylograms were constructed separately for each of the sequenced regions using BEAST v.1.8.3 (Drummond et al., 2012). The parameters of the nucleotide substitution models for each genomic regions analyzed were estimated during the analysis by BEAST, using the evolutionary model GTR + G as a prior. The Markov chain Monte Carlo runs with 30^8^ generations were performed with CIPRES v.3.3 (Miller, Pfeiffer & Schwartz, 2010) and an initial random tree as prior and the speciation model proposed by Yule (1925) and Gernhard, Hartmann & Steel (2008) were used. Data convergence was verified by Tracer v1.6 (Rambaut, Suchard & Drummond, 2013) and Tree Annotator v.1.8.3 (Drummond et al., 2012) was used to find the maximum clade credibility tree after a 10% burn-in of the sampled trees. The Bayesian species phylogram was constructed with BEAST v.1.8.3 (Drummond et al., 2012), using the ACT, GAP, ITS and β-TUB regions and the same parameters as in the previous analyses. *Monilochaetes infuscans* was used as an outgroup (Liu et al., 2016; Xavier et al., 2019)

A Neighbor-Net (Bryant & Moulton, 2004) was constructed based on the five sequenced regions using the “uncorrected P distance” in software SplitsTree v.4.14.2 (Huson & Bryant, 2006). To compare the genetic grouping of the species, a Bayesian cluster analysis was performed using BAPS v.6.0 (Corander et al., 2008). Values of possible groupings from K = 1 to K = 30 were tested, using the option “Clustering with linked loci” indicated for sequence data (Corander & Marttinen, 2006).

### AFLP markers

The AFLP markers were used to verify the phylogenetic relationships and to estimate the genetic distance of isolates collected in this study. The technique was applied according to Vos et al. (1995), with modifications as described by Cardoso et al. (2018).

In this case, for selective amplification, eight primer combinations were chosen to develop a fluorescent multiplex assay (*Eco*RI (FAM)/-AAC/*Mse*I-CT, *Eco*RI (NED)—ACA/*Mse*I-CTA, *Eco*RI (VIC)—ACT/*Mse*I-CA, *Eco*RI (PET)—AAG/*Mse*I-CAG, *Eco*RI (FAM)/-ACG/*Mse*I-CTAG, *Eco*RI (VIC)—ACT/*Mse*I-CAC, e *Eco*RI (NED)—AAC/*Mse*I-CATA, *Eco*RI (PET)—AGC/*Mse*I-CTCG). The results were combined in a binary matrix with software GeneMapper^®^ v. 4.1 (Applied Biosystems, Foster City, CA, USA).

### AFLP data analysis

For the AFLP data, a Bayesian cluster analysis was performed a mixture analysis on the software BAPS v.6.0 (Corander et al., 2008) to determine which genetic group the isolates belong to. Values of K = 1 to K = 15 possible clusters were tested using the “Clustering with linked loci” prior, indicated for the AFLP data (Corander & Marttinen, 2006). An admixture analysis was performed after the mixture analysis to calculate the ancestral genotype mixture of each fungal isolates from the genetic groups found (Corander & Marttinen, 2006).

A Nei-Li (Nei, 1972) pairwise distance matrix was calculated between isolates by the R package “poppr” (Kamvar, Brooks & Grünwald, 2015). Based on this matrix, a Principal Coordinate Analysis (PCoA) was performed using the “cmdscale” function and a dendrogram was constructed by the UPGMA clustering method. All analyses were implemented in the environment R v.3.4.1 (R Development Core Team, 2013). A *Fusarium* sp. sample was used as an outgroup to root the dendrogram.

### Microscopy of *Colletotrichum* spp. colonization on *Capsicum annuum*

The sweet pepper accession GB-103 (*C. annuum*) was chosen to visualize the colonization of UEL8.1U *Colletotrichum* spp. isolate. This isolate was preferred for being more aggressive than the others in previous studies of LMGV/UENF (De Almeida et al., 2020), while accession GB-103 is considered as a susceptibility reference (Baba et al., 2019). Ripe and unripe fruits of GB-103 were collected and superficially disinfected in 1% (v/v) sodium hypochlorite solution for 5 min, followed by three washes with distilled water for 1 min (Silva et al., 2014). Fourteen fruits were used for inoculum infection, and one fruit was used as control (inoculation simulation with sterile distilled water).

To prepare the inoculum, the isolate was cultivated on PDA culture medium at pH 7.0 and incubated in a growth chamber at 25 °C under a 12:12 h light:dark photoperiod until colony formation used for inoculum suspension. The center of the fruits was inoculated under laboratory conditions by the injection method, as described by Kanchana-udomkan, Taylor & Mongkolporn (2004), with a 1705 TLL micro-syringe (Hamilton, Bonaduz, Switzerland). A needle depth of 1 mm was fixed to ensure a constant inoculum volume and lesion size. A 1 × 10^6^ conidia/mL^−1^ suspension was prepared before inoculation, counted in a Neubauer chamber. After inoculation, the fruits were placed in a moist chamber and incubated in the dark for 24 h at 25 °C with subsequent 12:12 h light:dark cycles.

The inoculated area was photographed daily under a stereoscope (Luxeo 4D Labomed^®^, Orange, CA, USA) at two magnifications (0.8 and 2.0 (×10)) and the samples were immediately collected for microscopy. Sampling began 24 h after inoculation and lasted seven days. The samples were prepared at the Laboratory of Cell and Tissue Biology—UENF.

For light microscopy, fruit epidermis fragments (2–4 mm^2^) were taken from different depths, and fragments of twice the size for scanning microscopy. The initial sample preparation consisted of fixation in a solution composed of glutaraldehyde (0.2 mL), paraformaldehyde (0.5 mL), sodium cacodylate buffer (one mL pH 7.2) and 0.3 mL deionized water. Thereafter, the samples immersed in this solution were left to stand for 90 min at room temperature and then stored at 4 °C.

For processing, the samples were washed three times in one mL sodium cacodylate (0.05 M pH 7.2) for 45 min each at room temperature. Subsequently, the samples were post-fixed in a mixture of sodium cacodylate (0.1 M pH 7.2) plus 1:1 osmium tetroxide for 60 min in the dark. Thereafter, the samples were washed again three times in sodium cacodylate (0.05 M). The samples were dehydrated for 1 h per step in an increasing acetone concentration series (30, 50, 70, 90 and 100%).

### Optical microscopy

The 2–4 mm^2^ epidermis fragments were dried with acetone plus Epon—Epoxy resin at a 3:1 ratio, for 13 h. After this procedure, the tissue was infiltrated in 6 h intervals, at the following acetone proportions: Epon 2: 1; 1: 1; 1: 2; 1: 3 and 0: 3. The samples were embedded (polymerized) at 60 °C for 48 h. Paradermal and transverse epidermis sections (0.50 µM) were cut with an ultramicrotome (Reicheit Ultracut S). The sections were stained with 1% toluidine blue—sodium borate for 5 to 10 min, washed under tap water, dried on a hot plate and examined under a microscope (Zeiss, Jena, Germany). The images were captured with a 14 megapixel Cannon Power Shot camera and processed by Axiovision 4.8.

### Scanning electron microscopy (SEM)

Epidermal fragments (4–8 mm^2^) were immersed in 100% acetone and copper sulfate and dried to the critical point using 14 substitutions in liquid CO_2_ (Balzers CPD 030; BAL-TEC AG, Balzers, Liechtenstein). After dehydration, the samples were coated with a 10 nm gold-palladium (Au–Pd) film for 2 min. On each SEM stub, four samples were placed on carbon tape, two with a transverse view to the epidermis and two with superior view to the epidermis. The samples were examined under a scanning electron microscope (DSEM 962-ZEISS/Inspect 50-FEI) at 10–20 kV.

### Pathogenicity of UEL8.1U isolate against *Capsicum baccatum* accessions

The pathogenicity of UEL8.1U isolate was analyzed by an assessment of 50 *C. baccatum* accessions and one *C. annuum*, GB-103 accession. The *C. baccatum* accessions were from different regions of Brazil and had been described by Cardoso et al. (2018). The seeds were sown in 128 cell polystyrene trays containing organic plant substrate. After the emergence of two pairs of adult leaves, the seedlings were planted on an experimental field of the Universidade Estadual de Londrina (UEL) at a row and plant spacing of 1.0 m and 0.80 m, respectively. The field was managed according to the recommendations of cultural treatments for pepper cultivation.

The inoculation was performed with detached fruits as described by Silva et al. (2014). Six unripe and six ripe fruits per accession were randomly collected (35 and 50 days after anthesis (DAA), respectively) and superficially disinfected in 1% (v/v) sodium hypochlorite solution for 5 min, followed by three washes with distilled water for 1 min (Silva et al., 2014). Five fruits were used for infection with inoculum and one fruit was used as control (simulated inoculation with sterile distilled water). The inoculum was prepared following the same method as cited before to *C. annuum* microscopy analyses.

Lesion intensity was assessed on a 1–10 score scale every 24 h, between the 1^st^ and 8^th^ day after inoculation (DAI), as proposed by Montri, Taylor & Mongkolporn (2009) where: 1 = highly resistant; 3 = resistant; 4 = moderately resistant; 6 = moderately susceptible; 8 = susceptible; and 10 = highly susceptible. The periodic observation data were used to calculate the Area Under the Disease Progress Curve (AUDPC) (Campbell & Madden, 1990).

The data were subjected to non-parametric analysis of variance and the means compared by Dunnett’s test (*p* < 0.05). Spearman’s correlation was estimated between the variables Disease severity score and AUDPC at two fruit development stages (ripe and unripe). All analyses were implemented in software R v.3.4.1 (R Development Core Team, 2013) with the R package ‘agricolae’ (De Mendiburu & De Mendiburu, 2019).

## Results

### Morpho-cultural characterization of *Colletotrichum* spp. Isolates

**Figure 1:**
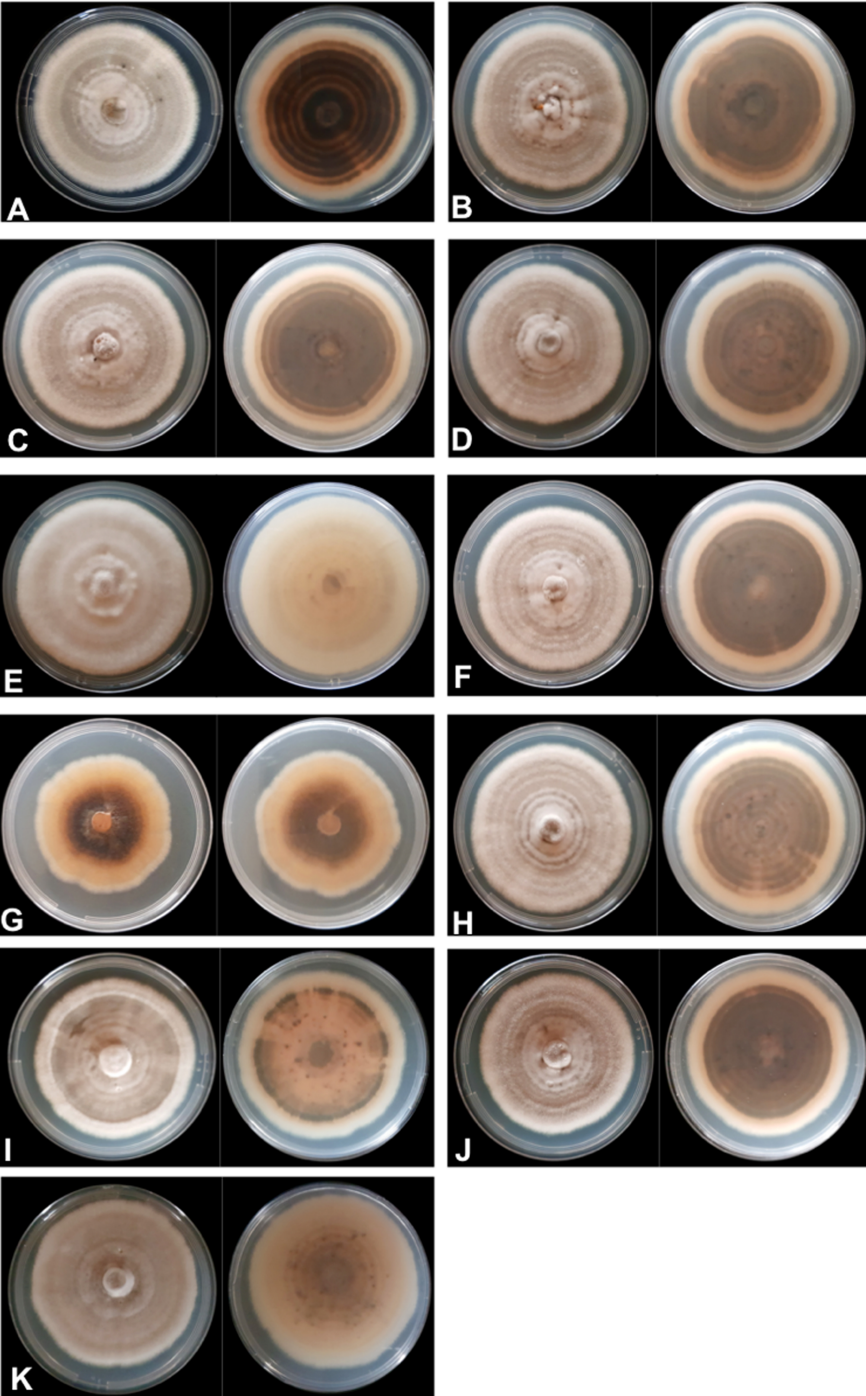
Mycelium appearance and color of 11 *Colletotrichum* spp. isolates. Upper and underside view of the plate with PDA culture medium (A) UEL01; (B) UEL09; (C) UEL8.1U; (D) UEL8.1F; (E) UEL12; (F) UEL22; (G) UEL27; (H) UEL42; (I) UEL53; (J) UEL71; (K) UEL72.

The mycelial and colony characteristics of 11 *Colletotrichum* isolates were analyzed after 10 days of isolate growth on PDA medium. The characteristics of most isolates, except UEL12 and UEL27, were similar. The mycelium of the nine isolates with similar characteristics formed moderately aerial, soft-looking, cottony colonies, with a whitish-gray upper colony surface and grayish brown on the underside, with concentric rings with a dark gray center. The conidial mass was salmon-colored (Fig. 1).

The growth characteristics of isolate UEL12 differed from those of the others. The aerial mycelium developed much more intensely, and the upper colony surface was also grayish, while no concentric rings were observed on the underside, which was grayish and brownish only in the center of the colony. On the other hand, a non-aerial mycelial growth of isolate UEL27 was observed, intensely salmon pink on the upper side, light brown on the underside and dark brown in the center. The colony margins were lighter colored, and no concentric rings were visible (Fig. 1). No significant differences in growth rates were observed among the 11 isolates on PDA medium. After 10 days of growth, the colony size varied from 43 to 82 mm, while the mean mycelium growth rate was 6 mm/day.

The characteristics of conidia morphology were recorded on the 15^th^ day of isolate growth on PDA medium. As for the cultures, the conidia characteristics were similar. The 50 conidia observed for each isolate were hyaline, straight, cylindrical shaped and mostly had one rounded and one more pointed end. However, the spearhead tip could not be observed on all conidia, possibly due to overlapping during analysis. The mean conidia length was 10.5–18 μM and width 3–4 μM (Table S1).

Identification and phylogenetic relationships of isolates. The results of molecular identification based on partial gene sequences together with the phylogenetic analysis of each gene region, especially GAPDH, allowed the identification of *Colletotrichum* spp. isolates. This identification was performed using two approaches: search in the “Basic Local Alignment Search Tool” (Altschul et al., 1990) and a search in the online tool “Q-bank fungi database” (Bonants, Edema & Robert, 2013). Both approaches indicated 100% identity with the *C. scovillei* species, being confirmed by multilocus phylogenetic analysis, which generated a species tree from the joint analysis of the the five sequenced regions data sets. The sequences of each region for each isolate have been deposited in GenBank. The accessions numbers are included in the Table S3. A phylogenetic tree was constructed for each gene region, using sequences from 34 GenBank accessions of representative species of the three major *Colletotrichum* complexes: *C. acutatum, C. gloeosporioides* and *C. truncatum*, plus three species outside the complexes, and one specie as outgroup (*Monilochaetes infuscans*) (species and GenBank accession number see Table S3; phylogenetic trees of each gene region see Figs. S1–S4).

**Figure 2:**
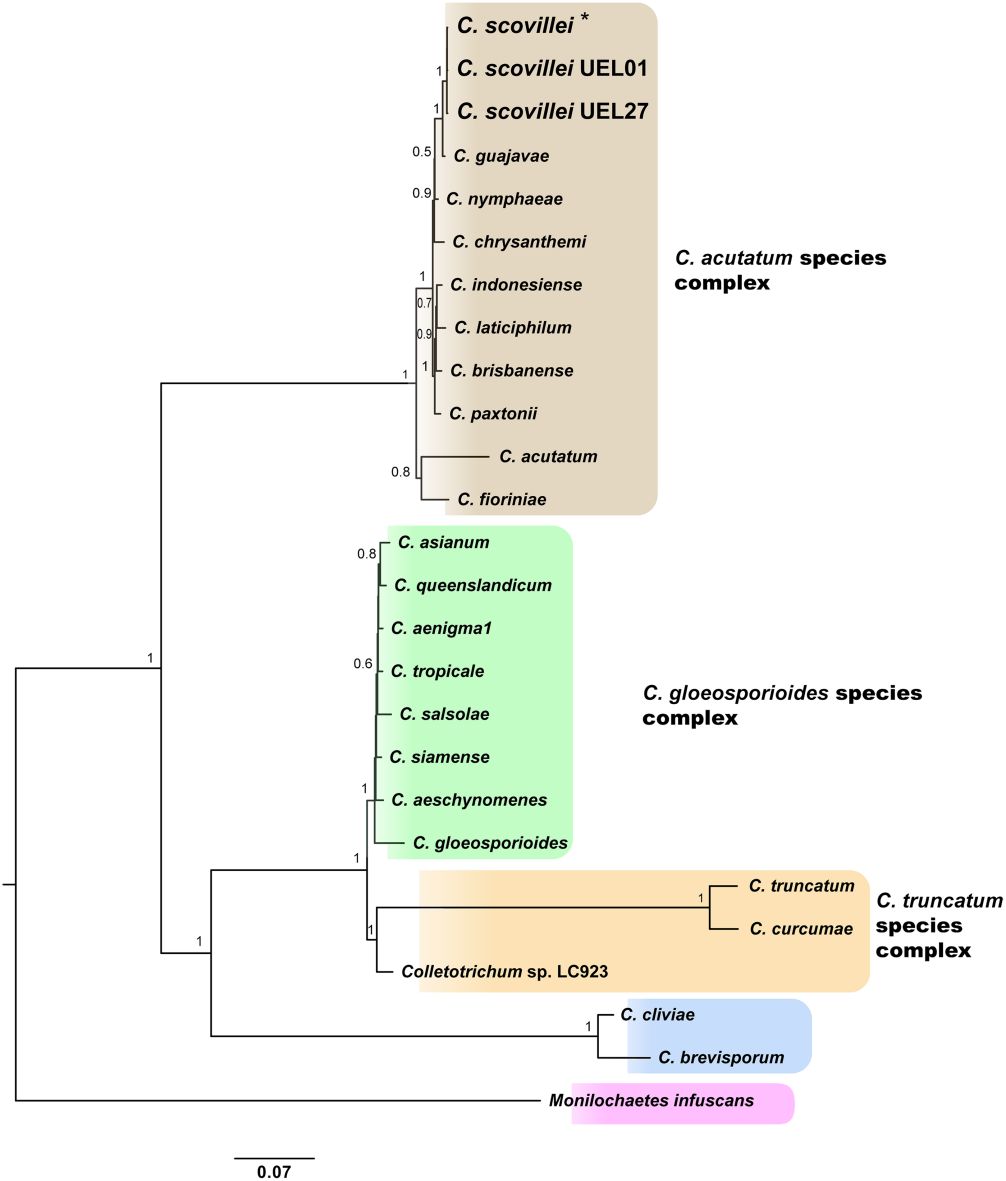
Bayesian phylogram of *Colletotrichum* species based on GAPDH, B-TUB, ACT, CAL and ITS gene regions inferred by BEAST. Numbers above branches represent Bayesian posterior probabilities (≥0.5). For the isolates used in this study, larger letters were used. *Indicates the *C. scovillei* accession from the GenBank. The scale bar (0.07) shows the number of substitutions per site. ****

**Figure 3:**
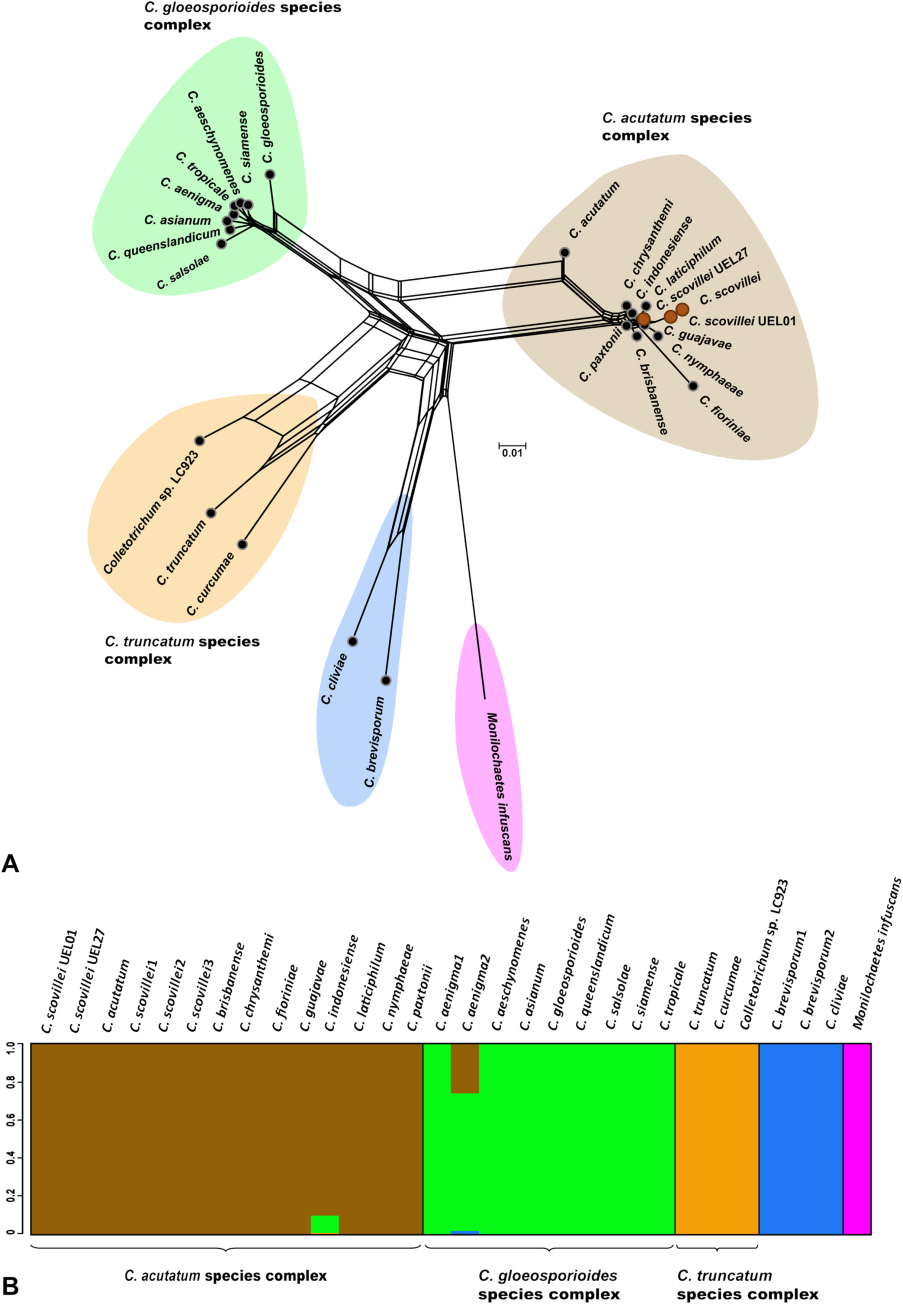
Phylogenetic relationships and Bayesian clustering of species of the genus *Colletotrichum* based on five genetic regions (GAPDH, B-TUB, ACT, CAL and ITS). (A) Neighbor-net constructed from the uncorrected P distance inferred by SplitsTree, and (B) Bayesian clustering analysis inferred by BAPS.

The Bayesian analysis grouped the *C. scovillei* isolates used in this study, together with a *C. scovillei* accession from the GenBank, into a subclade, within a clade with the other nine species of the *C. acutatum* complex (Fig. 2). The subclade formed by all *C. scovillei* samples presented maximum posterior probability (PP = 1) (Fig. 2). It was also observed the clustering of *Colletotrichum* species in five distinct groups, with maximum posterior probability (PP = 1). The first group was formed by the 10 species of the *C. acutatum* complex, where *C. guajavae* was associated (PP = 1) as sister species of *C. scovillei*. The second, third and fourth groups include the eight species of the *C. gloeosporioides* complex, two species of the *C. truncatum* complex, to which the *Colletotrichum* sp accession LC923 is grouped, and the species *C. cliviae* and *C. brevisporum*, whose complexes were not identified, respectively. As expected, *Monilochaetes infuscans*, a species close to *Colletotrichum* and a disease-causing pathogen in Solanaceae species, appears isolated as outgroup. A similar clustering pattern observed in the phylogram was also obtained using the Neighbor-Net method (Fig. 3A) and Bayesian clustering analysis (Fig. 3B).

### Variability of *Colletotrichum* *scovillei* isolates detected by AFLP markers

The sequencing of multigenic regions, together with the phylogenetic and morpho-cultural analyses, allowed the identification and classification of 11 *Colletotrichum* spp. isolates collected for this study. By this polyphasic analysis, all isolates could be classified as belonging to the *C. scovillei* Damm species, and differences between the phenotypic characteristics of the isolates were identified. Based on this result, the genetic variability among the isolates studied was estimated by AFLP markers. For this purpose, the technique was applied concomitantly to an external fungal isolate, in order to include an outgroup in the analyses.

The genetic variability among the isolates was estimated from data derived from eight selective AFLP primer combinations. The amplification products generated 694 fragments, distributed between 50 and 500 bp, of which 405 were polymorphic. The mean genetic distance, estimated by Nei’s coefficient (Nei & Li, 1979) between *C. scovillei* isolates, was 0.59. The shortest genetic distance was between isolates UEL8.1U and UEL22 and the longest between UEL8.1F and UEL12. In an overall comparison of all isolates, Nei’s distance for isolate UEL27 was the highest (distances between all isolates except the external group see Table S4).

**Figure 4:**
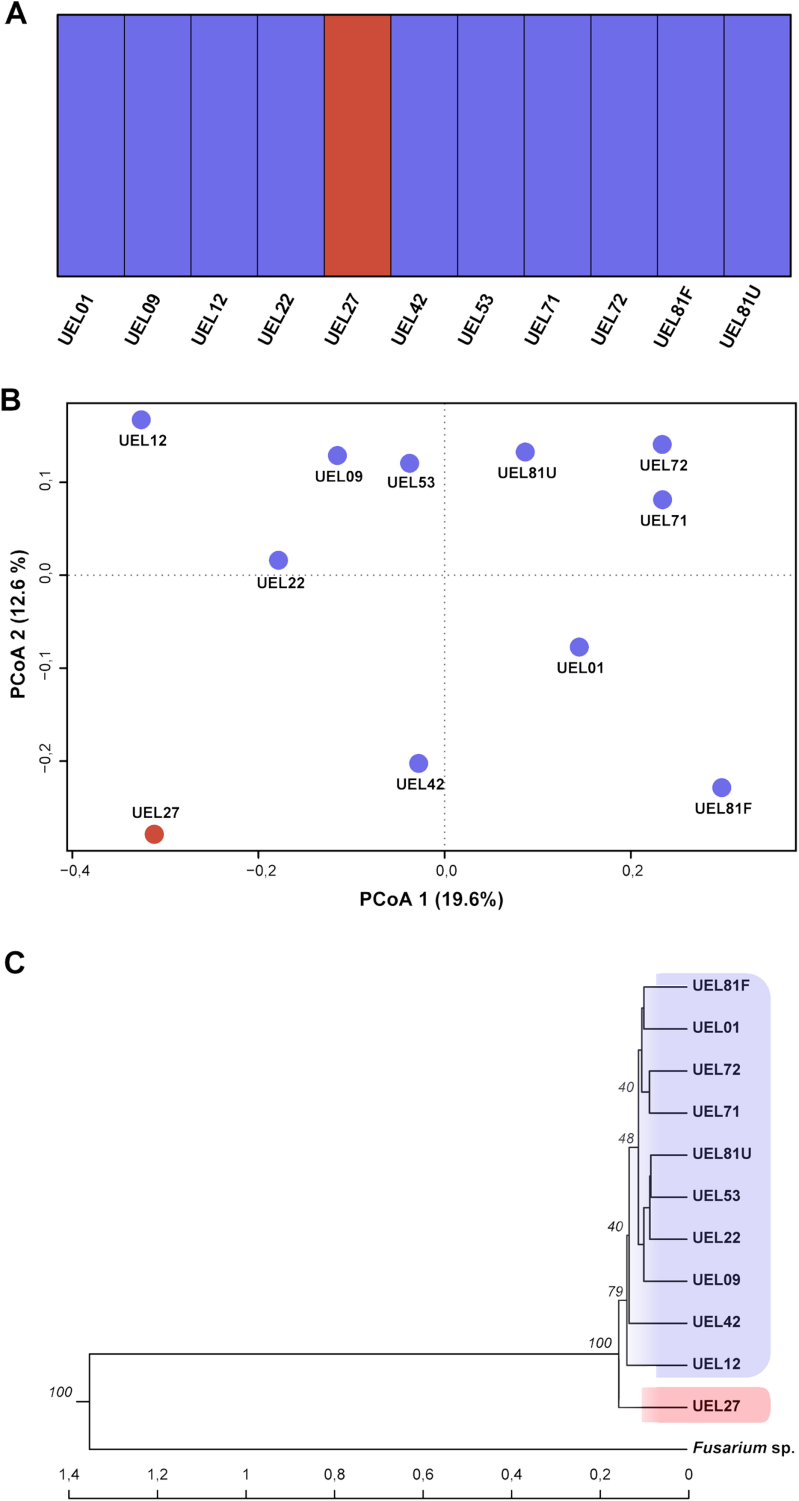
Cluster analysis based on 694 AFLP fragments showing the variability contained in the 11 *Colletotrichum scovillei* isolates. (A) Clusters formed by Bayesian genetic clustering analysis performed in BAPS; (B) Principal Coordinate Analysis (PCoA). (C) Dendrogram generated by UPGMA hierarchical clustering (sample *Fusarium* sp. was used as the outgroup to root the dendrogram).

The dendrogram established by UPGMA hierarchical clustering, when rooted with the outgroup, identified the formation of a single group with all *C. scovillei* isolates. In this group, isolate UEL27 is allocated more externally to the other samples, with a 100% bootstrap value (Fig. 4C). The possible number of clusters estimated by the Bayesian approach (excluding the external group, as similarly done with Nei’s distance) also identified a structure of isolate UEL27 that differed from the others. This analysis showed two groups in the set of isolates: one containing only isolate UEL27 and another with the other isolates (Fig. 4A). The principal coordinate analysis (PCoA) also confirmed the distance of isolate UEL27 from the others, by the graphical separation of the accession. The X and Y axes explained 32.2% of the variability of *C. sovillei* isolates (19.6% and 12.6% respectively) (Fig. 4B).

### Microscopic analysis of *Colletotrichum* *scovillei* colonization in unripe *Capsicum* *annuum* L. fruits

**Figure 5:**
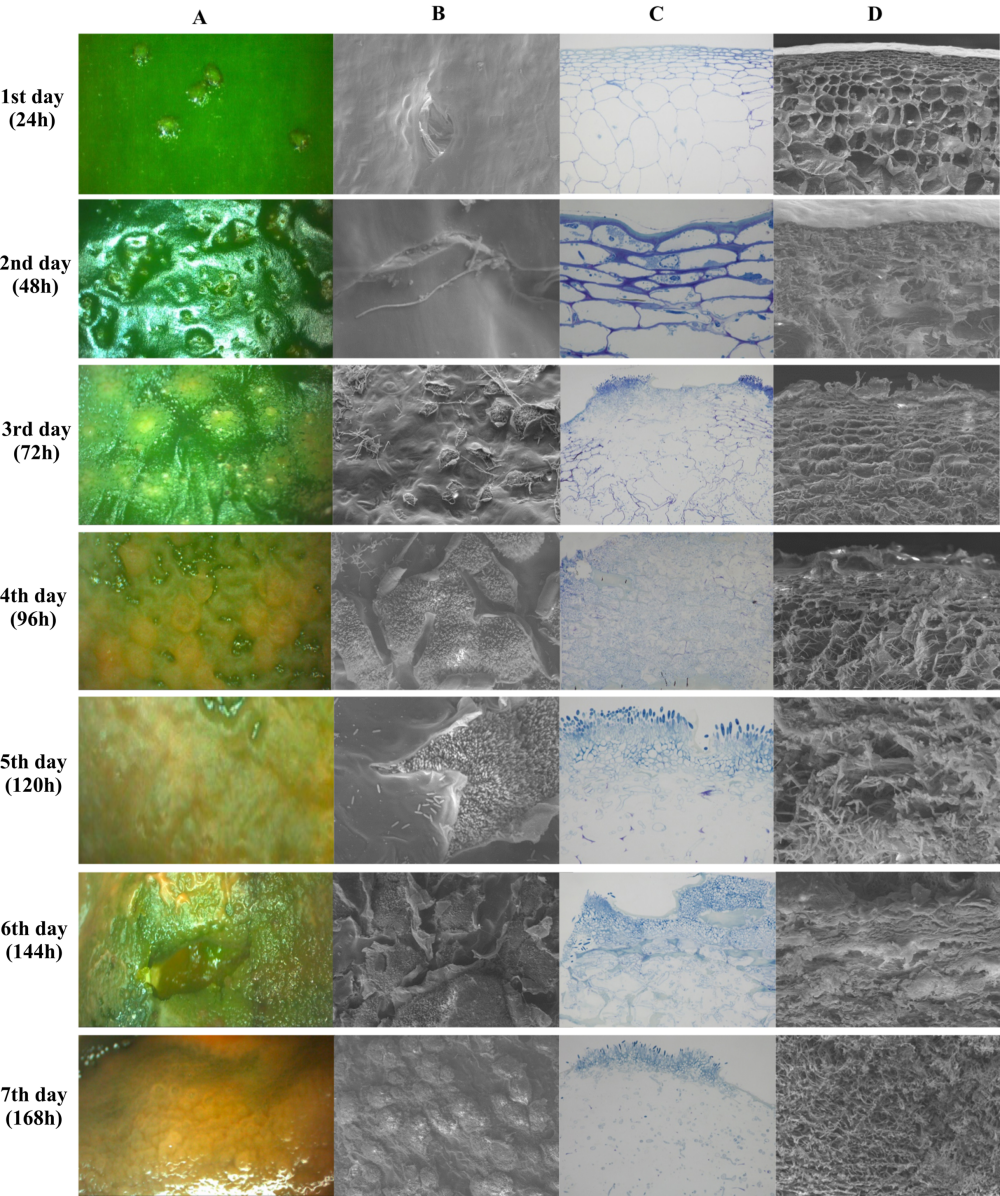
Microscopy images of infection and colonization of unripe *Capsicum annuum* fruits inoculated with *Colletotrichum scovillei*. Images were taken daily for seven days. In (A), electroscopic microscope images of the paradermic section of the inoculated region. In (B), a scanning microscope image of a paradigmatic section of the inoculated region. In (C), an optical microscope image of the transversal section and in (D) a SEM image of the transversal section.

Figure 5 shows images obtained by stereoscopic, optical and scanning electron microscopy (SEM) every 24 h after infection, for seven days. Figures 5A and 5B show the top view of the fruit epidermis in stereoscopic microscope and SEM images, respectively. Figures 5C and 5D are images of a transversal section of the epidermis in optical microscopy and SEM, respectively. The microscopic images show pathogen colonization over time, from spore germination until host cell degradation.

The incubation period of the disease in unripe fruits was 48 h or 2 DAI (Days After Inoculation), where small sunken spots in the epidermis and the beginning formation of colonization hyphae were observed. Optical microscopy images show the beginning of parenchymal cell colonization, with tissue depression caused by cell rupture. On the third day of infection, after 72 h, the stereomicroscope showed the presence of salmon-colored conidia, while the two transverse images showed the colonization and presence of hyphae already reaching the collenchyma. Optical microscopy images showed the presence of acervuli. Between the fourth and fifth day, germinated conidia were observed along the fruit epidermis and epidermis rupture with a cluster of acervuli. As of the sixth day of colonization, the epidermal cells were totally disintegrated in both the paradermic and transverse sections. On the last day of evaluation, after 168 h, conidia formation by secondary hyphae and total colonization and death of the host tissue were observed (Fig. 5).

**Figure 6:**
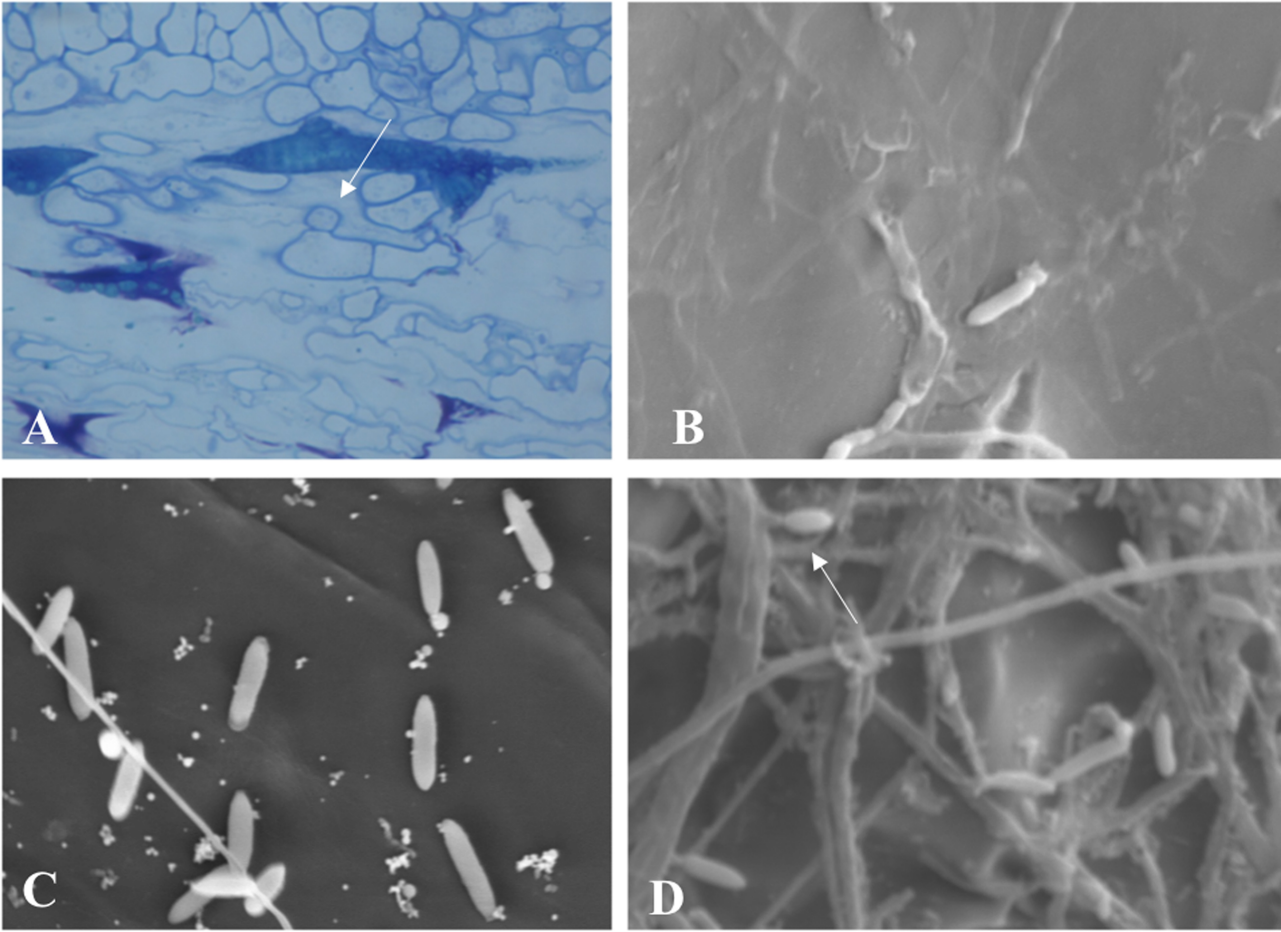
Microscopy images of *C. scovillei* colonization in *C. annuum*. (A) Germinated conidium with appressorium on the host surface. (B) and (C) Germinating conidia and (D) Conidia formation from secondary hyphae in a process of reinfection.

Throughout the periodic observations, colonization occurred according to the pattern described for the genus. Figure 6A shows conidia germination through a germ tube that gave rise to a globose cell called the appressorium from which the process of colonization and formation of primary hyphae is initiated. Figures 6B and 6C show images of conidia possibly in a process of reinfection as of the fourth or fifth day of colonization, soon after exposure of the conidial mass. Finally, Fig. 6D shows the exact moment of conidia formation from secondary hyphae on the epidermis of the infected fruit, an intrinsic characteristic of *C. scovillei* species. This process probably occurs as a kind of pathogen re-infection, no longer via the inoculum to which the fruit was exposed initially.

### Pathogenicity of *Colletotrichum scovillei* against *Capsicum* spp

**Figure 7:**
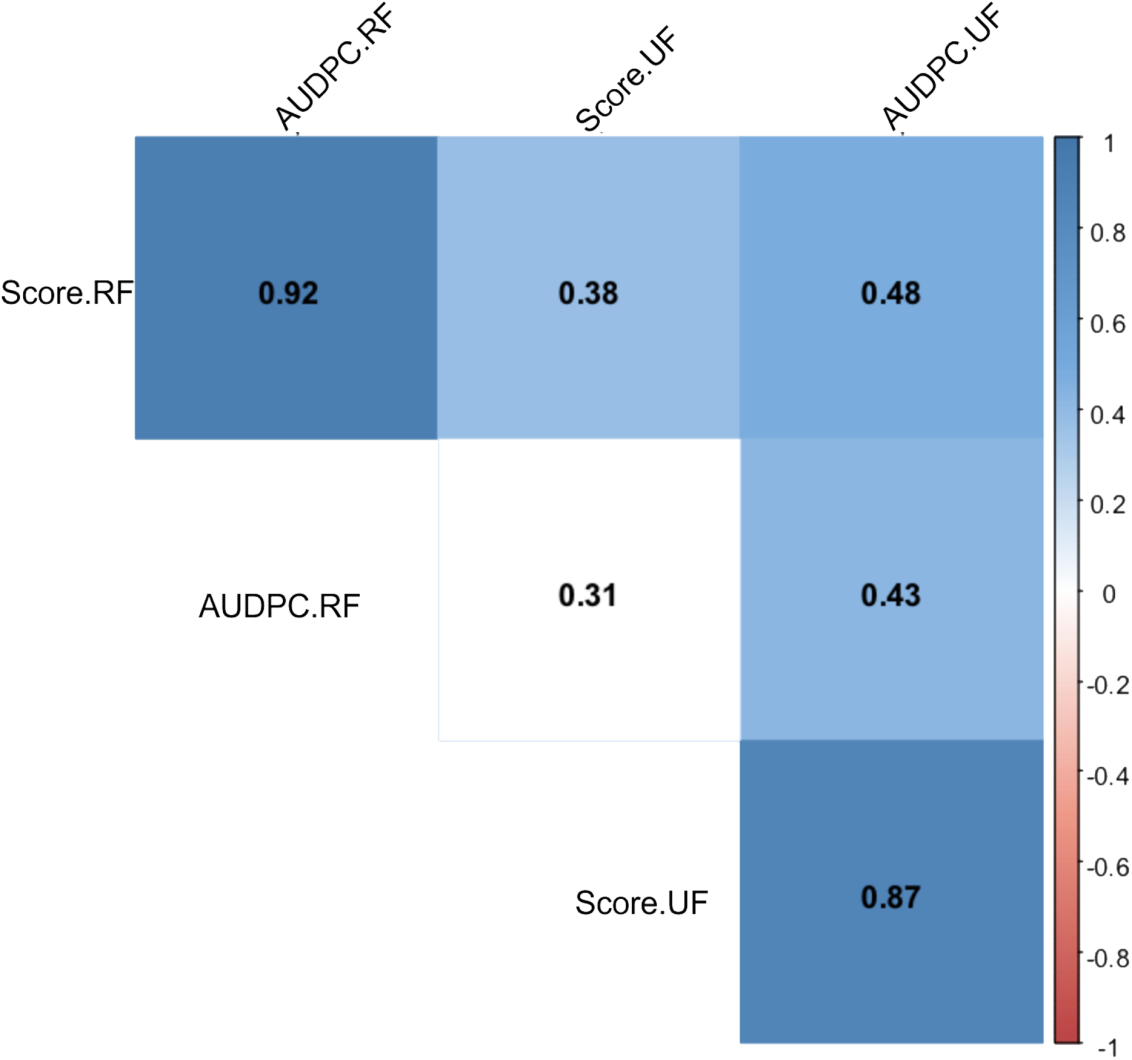
Spearman correlation between two variables: anthracnose severity score and Area Under the Disease Progress Curve—AUDPC in 51 *Capsicum* spp. accessions. UF, unripe fruits; and RF, ripe fruits.

After periodic evaluations every 24 h for eight days, different responses were observed in relation to the *Capsicum* species and fruit development stage. The nonparametric analysis of variance (ANOVA) showed a significant effect between the sources of variation: accessions, fruit development stage (FDS), interaction between FDS and mean disease severity score (Table 1). In general, higher anthracnose susceptibility was observed in unripe than in ripe fruits (mean final scores of 7.05 and 2.81 respectively, and mean AUDPC of 21.22 and 13.82, respectively) (Table 1). A high correlation was observed between AUDPC and disease score in ripe and unripe fruits (0.87 and 0.92, respectively) (Fig. 7). However, a low correlation was observed when the FDS were compared (Fig. 7).

**Table 1:** Non-parametric analysis and mean values for the effects of *Colletotrichum scovillei* inoculation on fruits of 51 *Capsicum* spp. accessions, at two stages of fruit development.

Sources of variation	ATS	GL	*p*-Value
**Disease severity score**			
Acessions (A)	7.88	29.72	<0.0001
Fruit development stages (FDS)	729.72	1.00	<0.0001
A × FDS	4.17	26.29	<0.0001
**AUDPC (Area Under the Disease Progress Curve)**			
Acessions (A)	1.91	27.92	0.003
Fruit development stages (FDS)	92.97	1.00	<0.0001
A × FDS	3.00	28.67	<0.0001
**Averages**	**Score**	**AUDPC**	
Unripe fruits	7.05	21.22	
Ripe fruits	2.80	13.82	

**Figure 8:**
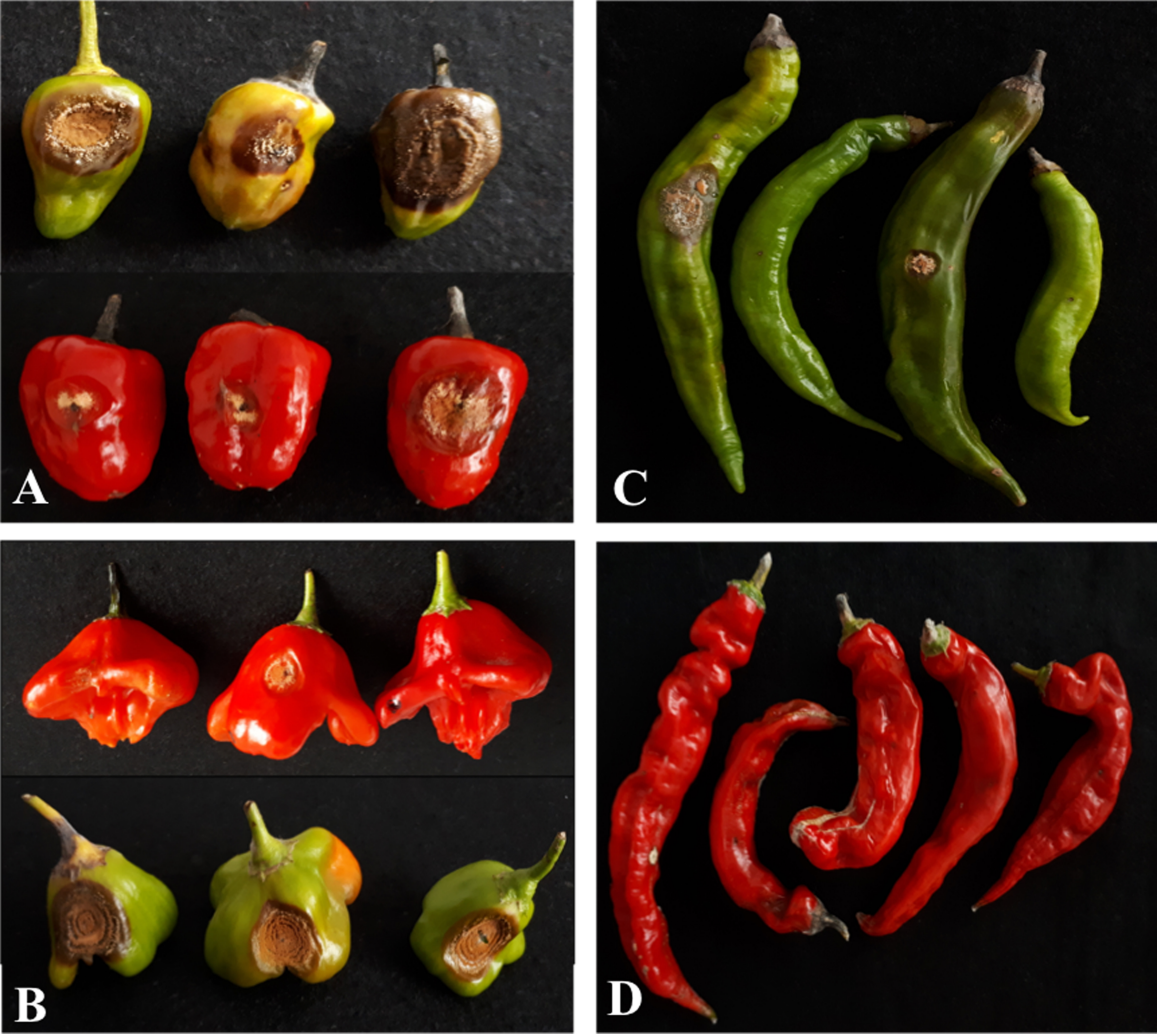
Pathogenicity of *Colletotrichum scovillei* on *Capsicum* spp. fruits seven days after inoculation. (A) GB-131; (B) GB-189; (C) GB-104 unripe fruits with anthracnose symptoms (D) GB-104 ripe fruits with no anthracnose symptoms.

Among the 51 studied accessions, 13 showed no significant differences by the Dunnett test (*p* < 0.05) with the lowest AUDPC values for unripe fruits and 37 for ripe fruits (Table 2). The accessions with a severity grade of ≤3 were considered resistant at both FDS. Resistance to *C. scovillei* at both FDS was observed in four accessions (GB-113, GB-118, GB-208 and GB-215). The disease susceptibility of both ripe and unripe *C. annuum* GB-103 fruits, used as susceptibility pattern, was confirmed (severity scores of 8.40 and 10, respectively). In general, typical lesions were observed in all *Capsicum* spp. accessions, differing only in the pathogenicity degree between accessions and FDS. Pictures of the typical disease symptoms on some accessions (Figs. 8A and 8B) also showed the difference in the reaction to the pathogen in relation to the FDS, showing symptoms on unripe fruits (Fig. 8C) and no symptoms on ripe fruits (Fig. 8D).

**Table 2:** Area under the disease progress curve (AUDPC) and disease severity score of *Colletotrichum scovillei* inoculation on fruits of 51 *Capsicum* spp. accessions, at two stages of fruit development stages.

Accessions	Unripe fruits			Ripe fruits		
	AUDPC	Score	Class^3^	AUDPC	Score	Class
GB-193	24.10	9.20	HS	9.80*	5.60	MS
GB-157	16.10*	7.20	S	16.30	5.60	MS
GB-189	26.50	10.00	HS	13.70	4.80	MS
GB-137	17.60	7.20	S	10.40	3.80	MR
GB-114	17.80	8.40	HS	8.00*	1.00	HR
GB-105	18.30	8.40	HS	9.40*	3.00	R
GB-145	19.50	8.40	HS	8.80*	3.60	MR
GB-214	13.50*	6.40	S	8.00*	1.80	R
GB-113	10.80*	2.60	R	8.00*	1.00	HR
GB-207	20.70	6.80	S	14.60	5.00	MS
GB-116	22.70	9.20	HS	19.70	8.40	HS
GB-212	18.30	7.60	S	9.80*	3.80	MR
GB-219	16.50	6.80	S	9.20*	2.20	R
GB-130	17.20	5.60	MS	11.60	3.80	MR
GB-110	23.70	10.00	HS	8.00*	1.00	HR
GB-136	18.00	5.40	MS	9.40*	3.00	R
GB-141	14.60*	5.20	MS	8.90*	2.60	R
GB-210	17.30	6.00	MS	8.00*	1.00	HR
GB-187	17.00	7.00	S	8.40*	2.20	R
GB-182	12.20*	4.20	MS	9.60*	1.80	R
GB-101	20.90	10.00	HS	8.20*	1.80	R
GB-190	19.30	6.80	S	8.20*	1.40	R
GB-216	17.10	8.00	S	8.20*	2.00	R
GB-111	18.30	6.00	MS	8.20*	1.60	R
GB-119	10.10*	3.40	MR	8.70*	2.80	R
GB-107	20.70	7.60	S	8.70*	1.60	R
GB-126	12.60*	3.80	MR	8.20*	1.60	R
GB-134	37.30	10.00	HS	12.30	4.80	MS
GB-163	17.90	6.00	MS	8.00*	1.00	HR
GB-104	16.70	7.20	S	9.60*	2.80	R
GB-208	11.20*	3.00	R	8.40*	2.20	R
GB-118	12.70*	2.80	R	8.00*	1.00	HR
GB-135	27.70	10.00	HS	8.60*	1.40	R
GB-200	20.90	8.00	S	8.00*	1.80	R
GB-172	27.30	9.20	HS	9.10*	3.20	MR
GB-184	23.70	9.20	HS	9.10*	2.40	R
GB-177	14.80*	5.00	MS	8.00*	1.80	R
GB-159	13.10*	5.20	MS	10.40	1.80	R
GB-103	12.00*	4.20	MS	8.00*	1.00	HR
GB-215^1^	8.90	2.60	R	8.00*	1.40	R
GB-156	16.10*	6.80	S	8.00*	1.00	HR
GB-223	20.50	7.60	S	8.20*	1.40	R
GB-146^2^	25.10	10.00	AS	8.00*	1.00	HR
GB-128	20.50	6.00	MS	17.30	6.00	MS
GB-129	29.70	10.00	HS	13.30	4.40	MS
GB-132	21.50	8.00	S	10.10	3.00	R
GB-150	23.50	8.80	HS	10.40	3.40	MR
GB-201	22.30	9.60	HS	9.50*	2.40	R
GB-178	17.00	5.00	MS	9.30*	2.20	R
GB-131	22.90	8.40	HS	14.70	6.00	MS
GB-103	31.46	10.00	HS	18.78	8.40	HS

**Notes:**

* Non-significant difference between control means by Dunnett’s test (*p* < 0.05).

1 Accession used as resistance control for unripe fruits.

2 Accession used as resistance control for ripe fruits.

3 Score scale proposed by Montri et al. (2009).

HR, highly resistant; R, resistant; MR, moderately resistant; MS, moderately susceptible; S, susceptible; HS, highly susceptible.

## Discussion

In this study, the association of morphological and molecular approaches was used to identify *Colletotrichum* isolates that induce typical anthracnose symptoms on pepper at two locations in the Rio de Janeiro state, with distinct topography and climate. An association of methodologies has been strongly recommended, since the stable and reliable molecular data can complement the morphological data which, in spite of the strong environmental influence, also characterize the species and contribute to their differentiation and identification (Cai et al., 2009; Liu et al., 2016; Mongkolporn & Taylor, 2018). By combining data of morphological analyses, e.g., of culture appearance and conidia morphology, and molecular analyses, e.g., sequencing of conserved regions in the fungal genome, the isolates of *Colletotrichum* spp. were identified and characterized as belonging to *C. scovillei* Damm species.

*Colletotrichum scovillei* belongs to the second clade of the *C. acutatum* complex. Although ITS sequences are considered as a barcode region for fungal identification, according to Sharma & Shenoy (2016), their sole use does not clearly identify the *acutatum* complex of *Colletotrichum*. On the other hand, the partial regions of the TUB2 and GAPDH genes are the most recommended for differentiation of this species (Damm et al., 2012). The above results were confirmed in our study, since the ITS region was one of the five with least variability among the species of the complex and the parsimony data of the GAPDH region was the highest. It is noteworthy that our results corroborated the literature in demonstrating that a polyphasic approach is the most appropriate (Damm et al., 2012; Oo et al., 2017). All morphological data reported here are in agreement with the descriptive characteristics of the species proposed by Damm et al. (2012).

*Colletotrichum scovillei* was first described in Thailand in 2008 (Than et al., 2008), and since then several studies identified the disease in areas of *Capsicum* spp. cultivation. It was also reported in Laos (Phoulivong et al., 2010), Japan (Kanto et al., 2014), China (Liu et al., 2016), and again in Thailand (Oo et al., 2017). In Brazil, it was first reported in 2014 on chili pepper, in the Minas Gerais state (Caires et al., 2014), and in 2017 in the states of Alagoas and Amazonas (Silva et al., 2017). However, studies on the characteristics, area of occurrence, management, control and resistance sources are incipient. In tests with *C. scovillei* against several *Capsicum* varieties, Oo et al. (2017) found no source of pathogen resistance.

Wide variability was observed among the isolates based on data from AFLP marker. Moreover, for isolate UEL27, relevant cultural differences were observed and it also proved to be genetically more distant from the others. Despite the low variation observed among the partial sequences of the genes studied in the 11 isolates, the observation of polymorphism through molecular markers evidence stout this studies are essential to understand the genetic relationships among isolates, as mentioned in the studies of Wijesekara, Aggarwal & Agarwal (2005) and Prittesh et al. (2016), who reported high genetic diversity within the *Colletotrichum* species.

A correct identification of the species, knowledge of pathogen variability and infection strategies are important steps to plan an efficient anthracnose control and management strategies for *Capsicum* spp. However, the most efficient way of reducing the damage caused by this disease is the use of resistant cultivars. Most of the commercially available pepper cultivars are susceptible to *Colletotrichum* spp. and, consequently, induce an excessive use of chemical fungicides for control (Saxena, Raghuwanshi & Singh, 2014; Ali et al., 2016). There are some resistance sources already described in the literature, but all of them are related to resistance to the most widespread species, such as *C. gloeosporioides*, *C. acutatum* and *C. capsici* (Pakdeevaraporn et al., 2005; Kim et al., 2008; Montri, Taylor & Mongkolporn, 2009; Silva et al., 2014), whereas few papers have been published on the *Capsicum* sp. × *C. scovillei* interaction. Therefore, it is important to examine the responses of accessions at local pepper cultivars to the most commonly found *Colletotrichum* species found in the region to provide genetic resources for pepper breeding programs.

To find these answers, one *C. scovillei* isolate (UEL8.1), previously selected for its virulence, was tested against 51 *Capsicum* spp. accessions in two fruit development stages. The variability of responses to *C. scovillei* and in relation to fruit development stages in the accessions was high. Some studies in the literature describe that different genes may confer anthracnose resistance according to the maturation stage. The studies of Pakdeevaraporn et al. (2005), Lin, Gniffke & Wang (2007), Than et al. (2008), Mahasuk et al. (2009), Bento et al. (2017) and Baba et al. (2019) and Giacomin et al. (2020) identified distinct genes responsible for resistance in ripe and unripe fruits, as well as different genes in relation to resistance to different pathogens. Similarly with the works cited, the ripe fruits were more resistant than unripe fruits. This higher resistance of ripe fruits is possibly related to a higher antioxidant production and concentration during fruit ripening of *Capsicum* spp., aside from the induction of the expression of some phenolic compounds responsible for infection responses (Kim et al., 2001; Mahasuk et al., 2009; Giacomin et al., 2020). Among these phenolic compounds possibly involved in the resistance of ripe fruits are pepper esterase (PepEST) and capsidiol, since they were reported as the main substance accumulated in the regions of *Colletotrichum* sp. infection (Kim et al., 2001; Mahasuk et al., 2009; Silva et al., 2014).

Four *Capsicum baccatum* accessions used in this study were resistant at both stages of fruit maturation. These accessions are considered promising and important for future studies related to resistance, such as genetic inheritance and resistance mapping, and can be used in breeding programs. The simultaneously inoculated *Capsicum annuum* accession proved completely susceptible to the disease. This accession was used as a susceptibility reference to monitor *C. scovillei* infection and colonization by microscopy over the course of time. This characterization is fundamental to visualize the changes in cell morphology and necrosis throughout the days of infection, to be able to outline strategies to block this process.

## Conclusions

Summing up, our results show that *C. scovillei* is damaging the pepper production and trade in the state of Rio de Janeiro, Brazil. The characterization and identification of isolates presented here is the first step towards understanding the epidemiology and developing effective anthracnose control strategies. Especially with regard to *Capsicum* spp., the best alternative is to invest in breeding programs to select and develop cultivars that are *Colletotrichum* spp. resistant at all maturation stages. To this end, further studies are needed to deepen the knowledge about the pathogen and investigate factors related to *Capsicum* resistance, e.g., by identifying new resistance sources and studying genetic inheritance.

## Supplemental Information

10.7717/peerj.10782/supp-1 Supplemental Information 1 Bayesian phylogram of *Colletotrichum* species based on ACT gene region inferred by BEAST. Numbers above branches represent Bayesian posterior probabilities (≥0.5). The isolates used in this study are highlighted in pink. The scale bar (0.06) shows the number of substitutions per site. The tree was rooted with *outgroup Monilochaetes infuscans*.

10.7717/peerj.10782/supp-2 Supplemental Information 2 Bayesian phylogram of *Colletotrichum* species based on GAPDH gene region inferred by BEAST. Numbers above branches represent Bayesian posterior probabilities (≥0.5). The isolates used in this study are highlighted in purple. The scale bar (0.07) shows the number of substitutions per site. The tree was rooted with *outgroup Monilochaetes infuscans*.

10.7717/peerj.10782/supp-3 Supplemental Information 3 Bayesian phylogram of *Colletotrichum* species based on ITS gene region inferred by BEAST. Numbers above branches represent Bayesian posterior probabilities (≥0.5). The isolates used in this study are highlighted in green. The scale bar (0.03) shows the number of substitutions per site. The tree was rooted with *outgroup Monilochaetes infuscans*.

10.7717/peerj.10782/supp-4 Supplemental Information 4 Bayesian phylogram of *Colletotrichum* species based on TUB2 gene region inferred by BEAST. Numbers above branches represent Bayesian posterior probabilities (≥0.5). The isolates used in this study are highlighted in old pink. The scale bar (0.05) shows the number of substitutions per site. The tree was rooted with *outgroup Monilochaetes infuscans*.

10.7717/peerj.10782/supp-5 Supplemental Information 5 GenBank accessions links.

10.7717/peerj.10782/supp-6 Supplemental Information 6 Raw data for Table 1 and 2.

10.7717/peerj.10782/supp-7 Supplemental Information 7 Morphological and cultural characterization of 11 isolates of *Colletotrichum* spp.

10.7717/peerj.10782/supp-8 Supplemental Information 8 Five region and genes used in the multilocus analysis of the isolates of *Colletotrichum* spp. with their respective primers.

10.7717/peerj.10782/supp-9 Supplemental Information 9 Species of the genus *Colletotrichum* used in this work with details of the isolates and GenBank accessions numbers of the five gene regions.

10.7717/peerj.10782/supp-10 Supplemental Information 10 Nei’s Genetic Distance among the 11 isolates of *Colletotrichum* spp.

## Additional Information and Declarations

### DNA Deposition

The following information was supplied regarding the deposition of DNA sequences: Data is available at GenBank: - ACT actin: MN121771, MN121772, MN121773, MN121774, MN121775, MN121776, MN121777, MN121778, MN121779, MN121780. - GAPDH glyceraldehyde 3-phosphate dehydrogenase: MN121781, MN121782, MN121783, MN121784, MN121785, MN121786, MN121787, MN121788, MN121789, MN121790, MN121791. - ITS rDNA spacer region: MN121792, MN121793, MN121794, MN121795, MN121796, MN121797, MN121798, MN121799, MN121800, MN121801, MN121802. - CAL calmodulin: MN121803, MN121804, MN121805, MN121806, MN121807, MN121808, MN121809, MN121810, MN121811. - TUB β-tubulin: MN121812, MN121813, MN121814, MN121815, MN121816, MN121817, MN121818, MN121819, MN121820, MN121821, MN121822.

### Data Availability

The following information was supplied regarding data availability: The raw measurements are available in the Supplemental Files.
