# Supplementary figures and images for: Phenotypic, molecular and pathogenic characterization of Colletotrichum scovillei infecting Capsicum species in Rio de Janeiro, Brazil
Source: PeerJ. 2021 Apr 27;9:e10782. doi: 10.7717/peerj.10782 (PMC8086587; doi:10.7717/peerj.10782)

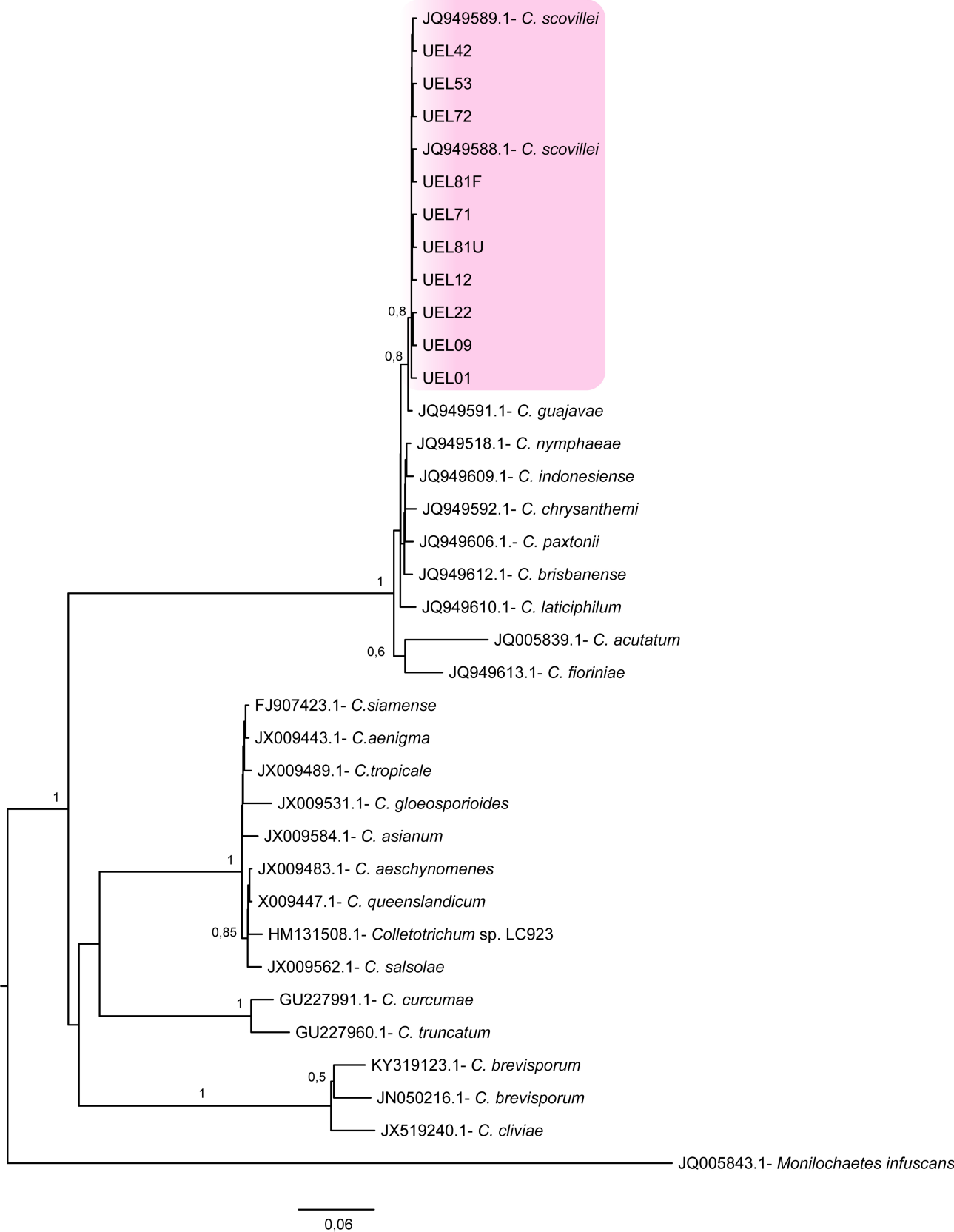

Supplement: Supplemental Information 1 — The scale bar (0.06) shows the number of substitutions per site. The tree was rooted with outgroup Monilochaetes infuscans. [file peerj-09-10782-s001.pdf]

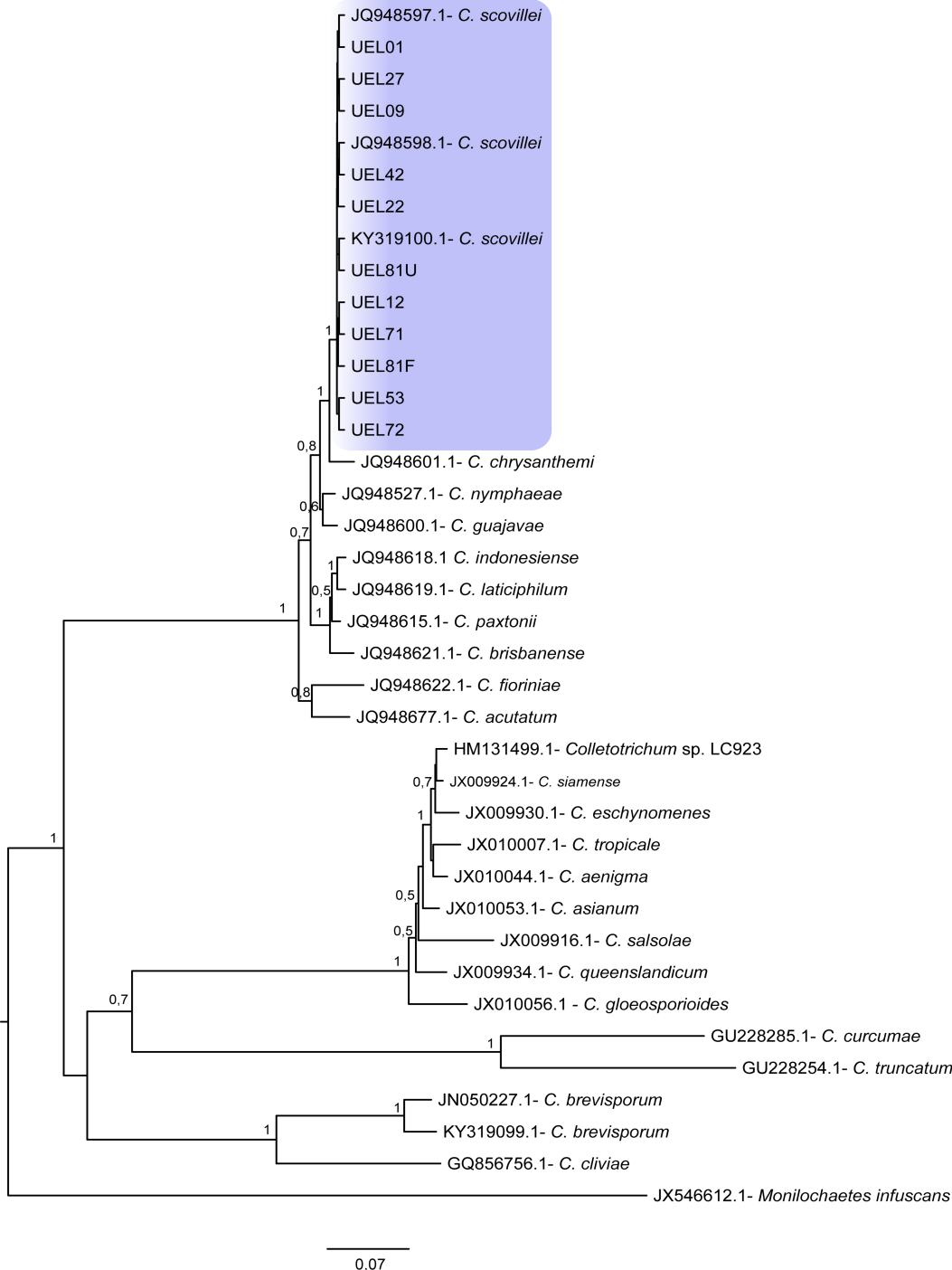

Supplement: Supplemental Information 2 — The isolates used in this study are highlighted in purple. The scale bar (0.07) shows the number of substitutions per site. The tree was rooted with outgroup Monilochaetes infuscans. [file peerj-09-10782-s002.pdf]

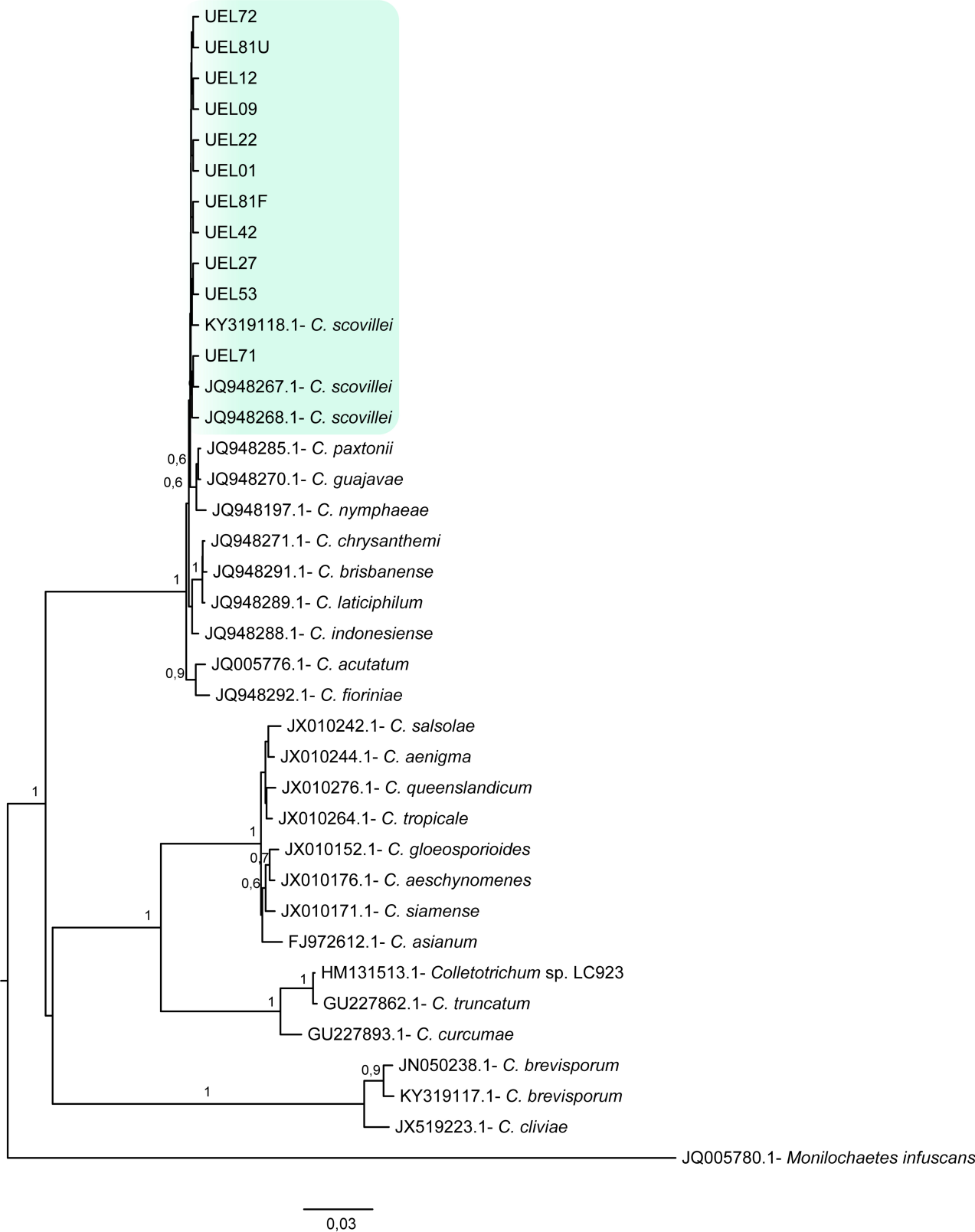

Supplement: Supplemental Information 3 — The isolates used in this study are highlighted in green. The scale bar (0.03) shows the number of substitutions per site. The tree was rooted with outgroup Monilochaetes infuscans. [file peerj-09-10782-s003.pdf]

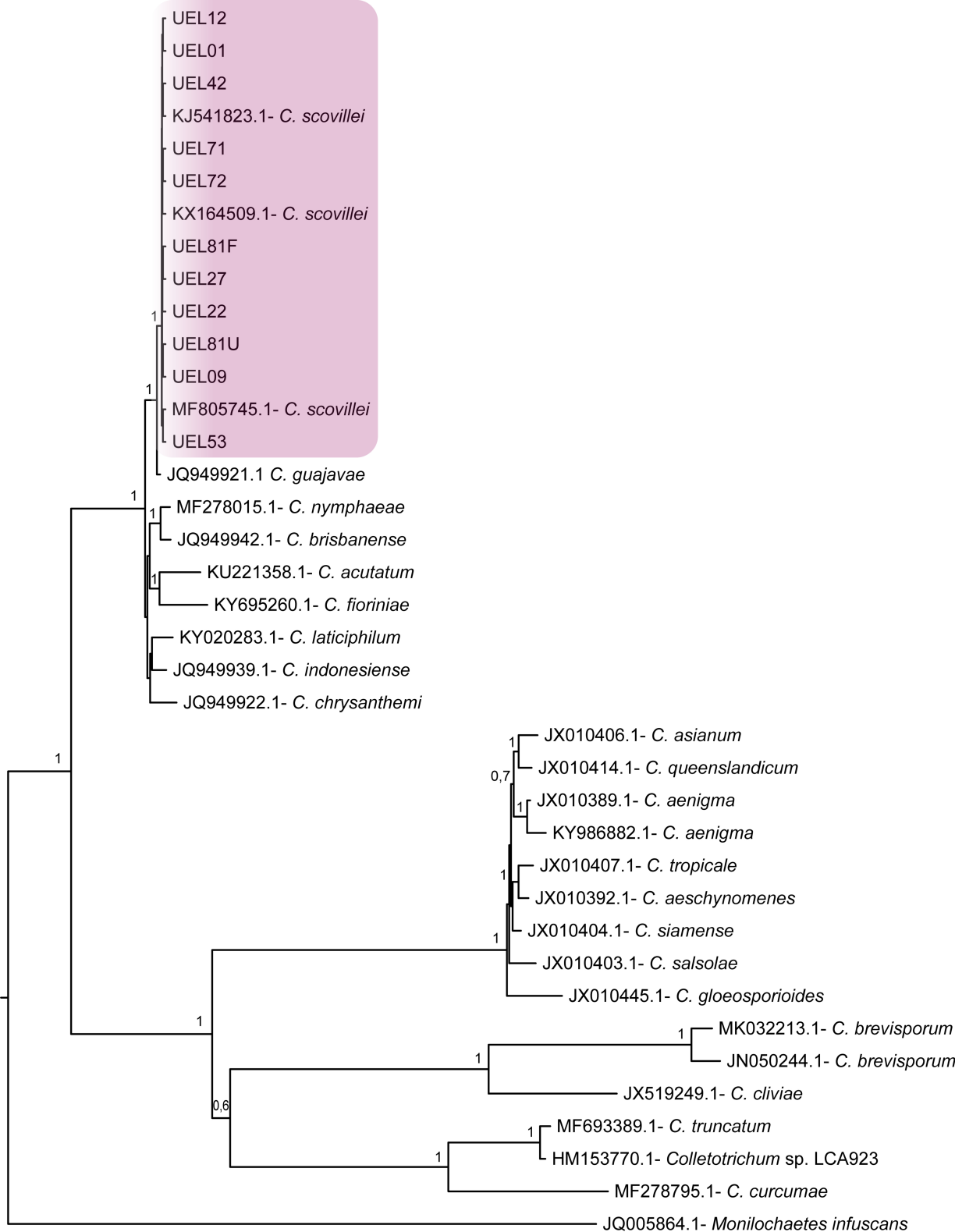

0,05

Supplement: Supplemental Information 4 — The isolates used in this study are highlighted in old pink. The scale bar (0.05) shows the number of substitutions per site. The tree was rooted with outgroup Monilochaetes infuscans. [file peerj-09-10782-s004.pdf]
