# Supplementary material for: Phenotypic, molecular and pathogenic characterization of Colletotrichum scovillei infecting Capsicum species in Rio de Janeiro, Brazil
Source: PeerJ. 2021 Apr 27;9:e10782. doi: 10.7717/peerj.10782 (PMC8086587; doi:10.7717/peerj.10782)
Supplement: Supplemental Information 7 [file peerj-09-10782-s007.docx]

**Supplementary table 1:** Morphological and cultural characterization of 11 isolates of *Colletotrichum* spp.

| **Identification** | **CGR** | **Mycelial appearance/color** | | **Conidia** | | **Origin** |
| --- | --- | --- | --- | --- | --- | --- |
|  |  | **Surface** | **Underside** | **Size (length vs width)(um)** | **Type** |  |
| UEL01 | 6.30 | moderately aerial, soft-looking, cottony colonies, with a whitish-gray. Conidial mass was salmon-colored | Grayish brown with concentric rings with a dark gray center. | 11-18 x 3 – 3.9 | hyaline, straight, cylindrical shaped and mostly had one rounded and one more pointed end. | Teresópolis-RJ |
| UEL8.1U | 5.80 | moderately aerial, soft-looking, cottony colonies, with a whitish-gray. Conidial mass was salmon-colored | Grayish brown with concentric rings with a dark gray center. | 12-19 x 5 – 6.9 | hyaline, straight, cylindrical shaped and mostly had one rounded and one more pointed end. | Nova Friburgo/RJ |
| UEL8.1F | 6.20 | moderately aerial, soft-looking, cottony colonies, with a whitish-gray. Conidial mass was salmon-colored | Grayish brown with concentric rings with a dark gray center. | 13 – 18 x 5 – 6 | hyaline, straight, cylindrical shaped and mostly had one rounded and one more pointed end. | Nova Friburgo/RJ |
| UEL09 | 6.20 | moderately aerial, soft-looking, cottony colonies, with a whitish-gray. Conidial mass was salmon-colored | Grayish brown with concentric rings with a dark gray center. | 10 – 17 x 3 – 4.1 | hyaline, straight, cylindrical shaped and mostly had one rounded and one more pointed end. | NI-1 |
| UEL12 | 6.40 | moderately aerial, soft-looking, cottony colonies, with a whitish-gray. Conidial mass was salmon-colored | Light gray and slightly asepsis in the center.. | 10.5 – 14 x 3.2 – 4.2 | hyaline, straight, cylindrical shaped and mostly had one rounded and one more pointed end. | São João da Barra-RJ |
| UEL22 | 6.10 | moderately aerial, soft-looking, cottony colonies, with a whitish-gray. Conidial mass was salmon-colored | Grayish brown with concentric rings with a dark gray center. | 10.5 – 12 x 3 – 4.4 | hyaline, straight, cylindrical shaped and mostly had one rounded and one more pointed end. | Campos dos Goyatacazes - RJ |
| UEL27 | 4.80 | Não aéreo, salmão e sépia ao centro. | Grayish brown with no concentric rings | 13 – 13.9 x 3 - 4 | hyaline, straight, cylindrical shaped and mostly had one rounded and one more pointed end. | Campos dos Goyatacazes - RJ |
| UEL42 | 6.00 | moderately aerial, soft-looking, cottony colonies, with a whitish-gray. Conidial mass was salmon-colored | Grayish brown with concentric rings with a dark gray center. | 11.9 – 14 x 4.1 - 5 | hyaline, straight, cylindrical shaped and mostly had one rounded and one more pointed end. | São João da Barra-RJ |
| UEL53 | 5.30 | moderately aerial, soft-looking, cottony colonies, with a whitish-gray. Conidial mass was salmon-colored | Grayish brown with concentric rings with a dark gray center. | 10.3 – 12 x 3 – 4.2 | hyaline, straight, cylindrical shaped and mostly had one rounded and one more pointed end. | Teresópolis – RJ |
| UEL71 | 6.20 | moderately aerial, soft-looking, cottony colonies, with a whitish-gray. Conidial mass was salmon-colored | Grayish brown with concentric rings with a dark gray center. | 11 – 18 x 3.2 – 3.9 | hyaline, straight, cylindrical shaped and mostly had one rounded and one more pointed end. | Teresópolis – RJ |
| UEL72 | 6.30 | moderately aerial, soft-looking, cottony colonies, with a whitish-gray. Conidial mass was salmon-colored | Grayish brown with concentric rings with a dark gray center. | 10.5 – 16 x 3 - 4 | hyaline, straight, cylindrical shaped and mostly had one rounded and one more pointed end | Teresópolis – RJ |
