# Supplementary material for: Phenotypic, molecular and pathogenic characterization of Colletotrichum scovillei infecting Capsicum species in Rio de Janeiro, Brazil
Source: PeerJ. 2021 Apr 27;9:e10782. doi: 10.7717/peerj.10782 (PMC8086587; doi:10.7717/peerj.10782)
Supplement: Supplemental Information 8 [file peerj-09-10782-s008.docx]

**Supplementary Table 2**: Five region and genes used in the multilocus analysis of the isolates of *Colletotrichum* spp. with their respective primers.

| **GENE** | **Final product** | ***Primer*** | ***Primer* sequence** | **Reference** |
| --- | --- | --- | --- | --- |
| GAPDH | Glyceraldehyde-3-phosphate desidrogenase | GD_F | GCCGTCAACGACCCCTTCATTGA | Templeton et al. (1992) |
|  |  | GD_R | GGGTGGAGTCGTACTTGAGCATGT |  |
| ITS | Internal transcribed spacer | ITS_F | CTTGGTCATTTAGAGGAAGTAA | White et al. (1990) |
|  |  | ITS_R | CTTGGTCATTTAGAGGAAGTAA |  |
| TUB2 | β -Tubulin | T1_F | AACATGCGTGAGATTGTAAGT | Glass & Donaldson (1995) |
|  |  | Bt2_F | GGTAAGCAAATCGGTGCTGCTTTC |  |
|  |  | Bt2_R | ACCCTCAGTGTAGTGACCCTTGGC |  |
| ACT | Actin | ACT512_F | ATGTGCAAGGCCGGTTTCGC | Carbone & Kohn (1999) |
|  |  | ACT783_R | TACGAGTCCTTCTGGCCCAT |  |
| CAL | Calmodulin | CL1_F | GAATTCAAGGAGGCCTTCTC | O’Donnel et al. (2000) |
|  |  | CL2_R | CTTCTGCATCATGACCTGGAC |  |
