# Supplementary material for: Phenotypic, molecular and pathogenic characterization of Colletotrichum scovillei infecting Capsicum species in Rio de Janeiro, Brazil
Source: PeerJ. 2021 Apr 27;9:e10782. doi: 10.7717/peerj.10782 (PMC8086587; doi:10.7717/peerj.10782)
Supplement: Supplemental Information 9 [file peerj-09-10782-s009.docx]

**Supplementary Table 3:** Species of the genus *Colletotrichum* used in this work with details of the isolates and GenBank accessions numbers of the five gene regions.

| Species from acutatum complex | Isolate code | Genbank accession number | | | | |  |
| --- | --- | --- | --- | --- | --- | --- | --- |
|  |  | **GAPDH** | **β-TUB** | **ACT** | **CAL** | **ITS** | |
| *C. acutatum* | CBS 112996; ATCC 56816/STE-U 5292; HTTJ3 | JQ948677 | JQ005860 | JQ005839 | MH113878 | JQ005776 | |
| *C. brisbanense* | CBS 292.67; DPI 11711 | JQ948621 | JQ949942 | JQ949612 | - | JQ948291 | |
| *C. chrysanthemi* | CBS 126518; PD 84/520 | JQ948601 | JQ949922 | JQ949592 | - | JQ948271 | |
| *C. fioriniae* | CBS 128517; LF603 | JQ948622 | JQ949943 | JQ949613 | KJ954727 | JQ948292 | |
| *C. guajavae* | CPC 18893; IMI 350839 | JQ948600 | JQ949921 | JQ949591 | - | JQ948270 | |
| *C. indonesiense* | CBS 127551; CPC 14986 | JQ948618 | JQ949939 | JQ949609 | - | JQ948288 | |
| *C. laticiphilum* | CBS 112989; IMI 383015, STE-U 5303 | JQ948619 | JQ949940 | JQ949610 | - | JQ948289 | |
| *C. nymphaeae* | CBS 515.78 | JQ948527 | JQ949848 | JQ949518 | - | JQ948197 | |
| *C. paxtonii* | IMI 165753; CPC 18868 | JQ948615 | JQ949936 | JQ949606 | - | JQ948285 | |
| *C. scovillei* | CBS 126529; PD 94/921-3, BBA 70349; HNCS015 | JQ948597 | JQ949918 | JQ949588 | KX673577 | JQ948267 | |
|  | CBS 126530; PD 94/921-4; LJTJ70 | JQ948598 | JQ949919 | JQ949589 | KP943584 | JQ948268 | |
| Supplementary table 3: Continued | COUFAL0054 | KY319100 | KY319109 | - | - | KY319118 | |
|  | UEL01 | MN121781 | MN121812 | MN121771 | MN121803 | MN121792 | |
|  | UEL09 | MN121782 | MN121813 | MN121772 | MN121804 | MN121793 | |
|  | UEL12 | MN121783 | MN121814 | MN121773 | MN121805 | MN121794 | |
|  | UEL22 | MN121784 | MN121815 | MN121774 | MN121806 | MN121795 | |
|  | UEL27 | MN121785 | MN121816 | MN121775 | MN121807 | MN121796 | |
|  | UEL42 | MN121786 | MN121817 | MN121776 | MN121808 | MN121797 | |
|  | UEL53 | MN121787 | MN121818 | MN121777 | MN121809 | MN121798 | |
|  | UEL71 | MN121788 | MN121819 | MN121778 | MN121810 | MN121799 | |
|  | UEL72 | MN121789 | MN121820 | MN121779 | MN121811 | MN121800 | |
|  | UEL81F | MN121790 | MN121821 | MN121780 | MN121812 | MN121801 | |
|  | UEL81U | MN121791 | MN121822 | MN121781 | MN121813 | MN121802 | |
| Species from  gloeosporioides complex | **Isolate code** | **Genbank acession number** | | | | | |
|  |  | **GAPDH** | **β-TUB** | **ACT** | **CAL** | **ITS** | |
| *C. aenigma* | ICMP 18608 | JX010044 | JX010389 | JX009443 | JX009683 | JX010244 | |
| *C. aeschynomenes* | ICMP 17673 | JX009930 | JX010392 | JX009483 | JX009721 | JX010176 | |
| *C. asianum* | ICMP 18580; CBS 130418 | JX010053 | JX010406 | JX009584 | FJ917506 | FJ972612 | |
| *C. gloeosporioides* | IMI 356878; ICMP 17821, CBS112999 | JX010056 | JX010445 | JX009531 | JX009731 | JX010152 | |
| *C. queenslandicum* | ICMP 1778 | JX009934 | JX010414 | JX009447 | JX009691 | JX010276 | |
| *C. salsolae* | ICMP 19051 | JX009916 | JX010403 | JX009562 | JX009696 | JX010242 | |
| *C. siamense* | ICMP 18578; CBS 130417 | JX009924 | JX010404 | FJ907423 | FJ917505 | JX010171 | |
| *C. tropicale* | CBS 124949; ICMP 18653, MTCC 11371 | JX010007 | JX010407 | JX009489 | JX009719 | JX010264 | |
| Species from truncatum complex | **Isolate code** | **Genbank acession number** | | | | | |
|  |  | **GAPDH** | **β-TUB** | **ACT** | **CAL** | **ITS** | |
| *C. truncatum* | CBS 151.35 | GU228254 | GU228156 | GU227960 | - | GU227862 | |
| *C. curcumae* | IMI 288937 | GU228285 | GU228187 | GU227991 | - | GU227893 | |
| *C. jasminigenum* | LC923 | HM131499 | HM153770 | HM131508 | HM131494 | HM131513 | |
| Other important species of the genus  *Colletotrichum* | **Isolate code** | **Genkank acession number** | | | | | |
|  |  | **GAPDH** | **β-TUB** | **ACT** | **CAL** | **ITS** | |
| *C. brevisporum* | BCC 38876 | JN050227 | JN050244 | JN050216 | - | JN050238 | |
|  | COUFAL0053 | KY319099 | KY319108 | KY319123 | KY319126 | KY319117 | |
| *C. cliviae* | CBS 125375 | GQ856756 | JX519249 | JX519240 |  | GQ849464 | |
| Outgroup |  |  |  |  |  |  | |
| *Monilochaetes infuscans* | CBS 869.96 | JX546612 | JQ005864 | JQ005843 |  | - | |

Supplementary table 3: Continued
