# Supplementary material for: Phenotypic, molecular and pathogenic characterization of Colletotrichum scovillei infecting Capsicum species in Rio de Janeiro, Brazil
Source: PeerJ. 2021 Apr 27;9:e10782. doi: 10.7717/peerj.10782 (PMC8086587; doi:10.7717/peerj.10782)
Supplement: Supplemental Information 10 [file peerj-09-10782-s010.docx]

**Supplementary Table 4:** Nei’s Genetic Distance among the 11 isolates of *Colletotrichum* spp.

|  | **UEL01** | **UEL09** | **UEL12** | **UEL22** | **UEL27** | **UEL42** | **UEL53** | **UEL71** | **UEL72** | **UEL81F** |
| --- | --- | --- | --- | --- | --- | --- | --- | --- | --- | --- |
| **UEL01** |  |  |  |  |  |  |  |  |  |  |
| **UEL09** | 0.57 |  |  |  |  |  |  |  |  |  |
| **UEL12** | 0.84 | 0.53 |  |  |  |  |  |  |  |  |
| **UEL22** | 0.52 | 0.42 | 0.51 |  |  |  |  |  |  |  |
| **UEL27** | 0.85 | 0.72 | 0.68 | 0.54 |  |  |  |  |  |  |
| **UEL42** | 0.65 | 0.64 | 0.79 | 0.59 | 0.83 |  |  |  |  |  |
| **UEL53** | 0.62 | 0.50 | 0.60 | 0.41 | 0.74 | 0.56 |  |  |  |  |
| **UEL71** | 0.63 | 0.57 | 0.89 | 0.64 | 0.94 | 0.69 | 0.66 |  |  |  |
| **UEL72** | 0.58 | 0.67 | 0.87 | 0.70 | 0.90 | 0.82 | 0.54 | 0.50 |  |  |
| **UEL81F** | 0.56 | 0.75 | 1.00 | 0.73 | 0.98 | 0.71 | 0.73 | 0.60 | 0.66 |  |
| **UEL81U** | 0.44 | 0.41 | 0.58 | 0.38 | 0.81 | 0.58 | 0.42 | 0.42 | 0.44 | 0.52 |
